# Nutraceuticals as Supportive Therapeutic Agents in Diabetes and Pancreatic Ductal Adenocarcinoma: A Systematic Review

**DOI:** 10.3390/biology12020158

**Published:** 2023-01-19

**Authors:** Iveta Mikolaskova, Tatjana Crnogorac-Jurcevic, Bozena Smolkova, Luba Hunakova

**Affiliations:** 1 Institute of Immunology, Faculty of Medicine, Comenius University, Odborarske Namestie 14, 811 08 Bratislava, Slovakia; 2 Barts Cancer Institute, Barts and The London School of Medicine and Dentistry, Queen Mary University, Charterhouse Square, London EC1M 6BQ, UK; 3 Biomedical Research Center, Slovak Academy of Sciences, Cancer Research Institute, Dubravska Cesta 9, 845 05 Bratislava, Slovakia

**Keywords:** diabetes, pancreatic ductal adenocarcinoma, curcumin, thymoquinone, genistein, *Ginkgo biloba*, low-carbohydrate ketogenic diet

## Abstract

**Simple Summary:**

Pancreatic ductal adenocarcinoma is one of the most lethal diseases, with an exceptionally poor prognosis. The successful clinical management of strongly related diabetes could significantly contribute to more efficient control of cancer development and progression. In this regard, various natural products have been explored. This review evaluates the therapeutic potential of four natural products (Curcumin—*Curcuma longa* L.; Thymoquinone—*Nigella sativa* L.; Genistein—*Glycine max* L.; *Ginkgo biloba* L.) and one nutritional intervention, in the form of a low-carbohydrate ketogenic diet in pancreatic cancer and diabetic patients, and discusses their possible integration in supportive cancer management. Although the results have shown their effectiveness in the treatment of diabetes, the therapeutic response and survival time were not significantly improved in pancreatic cancer patients, despite improvements in several biological parameters. Nevertheless, based on published data, the studied natural products and nutritional intervention can potentially become promising therapeutic approaches for pancreatic cancer risk reduction through early intervention at the onset of diabetic complications.

**Abstract:**

The correlation between pancreatic ductal adenocarcinoma (PDAC) and diabetes-related mechanisms support the hypothesis that early therapeutic strategies targeting diabetes can contribute to PDAC risk reduction and treatment improvement. A systematic review was conducted, using PubMed, Embase and Cochrane Library databases, to evaluate the current evidence from clinical studies qualitatively examining the efficacy of four natural products: Curcumin—*Curcuma longa* L.; Thymoquinone—*Nigella sativa* L.; Genistein—*Glycine max* L.; *Ginkgo biloba* L.; and a low-carbohydrate ketogenic diet in type 2 diabetes (T2D) and PDAC treatment. A total of 28 clinical studies were included, showing strong evidence of inter-study heterogeneity. Used as a monotherapy or in combination with chemo-radiotherapy, the studied substances did not significantly improve the treatment response of PDAC patients. However, pronounced therapeutic efficacy was confirmed in T2D. The natural products and low-carbohydrate ketogenic diet, combined with the standard drugs, have the potential to improve T2D treatment and thus potentially reduce the risk of cancer development and improve multiple biological parameters in PDAC patients.

## 1. Introduction

Pancreatic ductal adenocarcinoma (PDAC), representing more than 90% of all pancreatic cancers, is one of the deadliest cancers, with more than 495,000 cases diagnosed worldwide (2020). The majority of PDAC cases (80–90%) are diagnosed at a late, non-resectable stage, with a 5-year survival rate of just 5–10%. If the disease is metastatic, the average survival time is 3–6 months; the survival rate has shown little improvement over recent decades. The mortality almost equates to the incidence rate, with more than 466,000 PDAC deaths per year worldwide [1,2]. 

Due to the late manifestation of clinical symptoms, the only curative treatment for PDAC is surgery, which is indicated only in 10–15% of cases. The remaining 85% of individuals are those with unresectable local or metastatic disease [3]. Chemotherapy and radiotherapy are common treatments for the palliative management of the disease. Long-term survival remains rare, even in individuals undergoing resection [3]. 

A search for improved therapeutic strategies is an area of active basic, pre-clinical and clinical research.

### 1.1. Diabetes as a Risk Factor for PDAC Development

Several risk factors have been associated with the initiation and development of PDAC, including genetic susceptibility, chronic pancreatitis, dietary factors, being overweight or obese, microbiota imbalance, deficiency of 25-Hydroxyvitamin-D, as well as smoking, alcohol consumption, and sustained psychologic stress [4,5,6]. A well-known risk factor for PDAC is type 2 diabetes (T2D). Long-standing T2D carries an almost two-fold increased risk of PDAC. Approximately 85% of individuals diagnosed with PDAC experience impaired glucose tolerance (GT) or T2D [7]. Hyperglycemia-induced higher insulin secretion and hyperinsulinemia are implicated in T2D and cause insulin resistance (IR). [8,9]. Almost half of PDAC patients were diagnosed with new-onset diabetes that preceded cancer diagnosis by 2–3 years. This new-onset diabetes (type 3c diabetes), considered to be PDAC-associated diabetes, is related to exocrine pancreatic diseases, such as chronic pancreatitis, pancreatic neoplasms (PanIN), or pancreatic trauma. Patients with coexisting T2D and chronic pancreatitis have an increased risk (~up to eight-fold) of developing PDAC [10]. There are few studies evaluating PDAC incidence in type 1 diabetes (T1D). A systematic review of the risk of pancreatic cancer in people with T1D and young-onset diabetes suggests a similar association as in T2D. However, conclusions are limited, due to the scarcity of published data [11]. 

**Figure 1:**
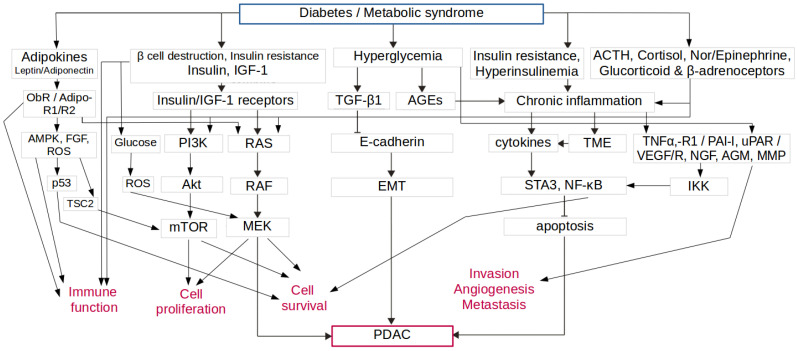
Molecular mechanisms associated with diabetes and pancreatic ductal adenocarcinoma (PDAC). ObR: leptin receptor; Adipo-R1/R2: adiponectin receptor 1/2; AMPK: adenosine monophosphate protein-activated kinase; TSC2: Tuberin, controlling the mammalian target of rapamycin (mTOR) signaling; IGF-1: insulin-like growth factor-1; ROS: reactive oxygen species; PI3K: phosphatidylinositol 3-kinase; MAPK: mitogen-activated protein kinase(Ras/Raf/MEK/ERK pathway); TGF-β1: transforming growth factor-β1; AGEs: advanced glycation end products; EMT: epithelial-mesenchymal transition; TME: tumor microenvironment; STAT3: signal transducer and activator of transcription 3; NF-κB: nuclear factor kappa B; ACTH: adrenocorticotropic hormone; TNF-α: tumor necrosis factor- α; TNF-R1: tumor necrosis factor-receptor 1; PAI-1: plasminogen activator inhibitor-1; uPAR: urokinase-type plasminogen activator receptor; VEGF: vascular endothelial growth factor; VEGFR: vascular endothelial growth factor receptor; IKK: IκA;B kinase/enzyme complex involved in the cellular response to inflammation; FGF: fibroblast growth factor; NGF: nerve growth factor; AGM: axonal guidance molecule; MMP: matrix metalloproteinases. Modified according to [7,15,16].

A meta-analysis and pooled-data studies have demonstrated a positive aetiologic correlation between T2D and PDAC [9,12] and the role of T2D and hyperinsulinemia in carcinogenesis [13]. As 80% of people with T2D are overweight/obese, obesity has been studied for its correlation with cancer. It has been shown that high BMI (>30) or obesity is positively associated with PDAC, as well as other types of cancer, including liver or colorectal cancer [14]. There are more possible mechanisms involved (Figure 1), but hyperglycemia, IR and related hyperinsulinemia, nutritional factors, obesity-related adverse effects, and inflammatory responses are potential causal factors for PDAC development [8]. 

IR causes insulin oversecretion, β-cell overactivity and increased β-cell mass. The tissue of the exocrine pancreas becomes chronically exposed to elevated levels of secreted insulin [15]. Its mitogenic activity promotes cell proliferation and growth, increases utilization of glucose, and thus contributes to the development of a tumor and its progression. Insulin also increases the bioavailability of IGF-1 [17]. IGF-1 exhibits substantial mitogenic and anti-apoptotic effects and, furthermore, potentiates the growth of insulin and insulin receptor-expressing cells. IGF-1 and its receptor are over-expressed in PDAC cells and enhance their proliferation, invasion, angiogenesis, and inhibition of apoptosis [18]. The binding of insulin and IGF-1 to their receptors initiates signal transduction that activates MAPK (Ras-Raf-MEK-ERK) and PI3K/Akt/mTOR pathways, promotes proliferation, and downregulates apoptosis [15]. Hyperglycemia, attributed to excess carbohydrate availability, glycation, and impaired detoxification, upregulates the formation of free radicals and advanced glycation end products (AGEs) and increases inflammation [19]. AGEs upregulate AGEs receptor in PanIN and stimulate PDAC invasiveness [20]. Hyperglycemia might also be responsible for acquiring mesenchymal and cancer stem cell features necessary for tumor initiation and progression. It is mediated by the hyperglycemia-activated TGF-β signaling that might provide another explanation for T2D facilitating PDAC [7].

Hyperinsulinemia and IR, associated with increased adiposity, activate a plethora of inflammatory cells, and induce systemic inflammation that contributes to genomic aberrations and tumorigenesis [21]. Pro-inflammatory cytokines (adipocytokines), such as leptin, adiponectin, tumor necrosis factor-alpha (TNF-α) and interleukin-6 (IL-6), are involved in immune responses, inflammation, apoptosis, and metabolism. They increase IR and may trigger malignant transformation, angiogenesis, tumor growth, migration, and metastasis [21]. Pro-inflammatory cytokines, reactive oxygen species (ROS), and inflammatory pathway players, including COX-2 or NF-κB, contribute to DNA damage, genomic instability and mutations that prime carcinogenesis, suppression of apoptosis, immunosuppression, inhibition of DNA repair, and stimulation of the cell cycle. Inflammation also affects TME by immune cells releasing cytokines and growth factors that promote tumor growth [7,22].

Hyperglycemia is also associated with neural invasion and increased secretion of nerve growth factor (NGF) that ensure a loop of further neural infiltration and tumor growth [23,24]. In addition, immune cells express β-adrenoceptors and glucocorticoid receptors. Therefore, there is a direct correlation between excessive stress hormones (catecholamines and cortisol) during chronic exposure to stress and suppressing immune and inflammation surveillance. In concurrent obesity, stress hormones-stimulated β-adrenoceptors trigger the release of pro-inflammatory complexes by neutrophils, the oxidation of lipids, the upregulation of fibroblast growth factors, and the activation of mitogenic signaling and proliferation of dormant tumor cells [5]. 

### 1.2. Nutraceuticals-Based Treatment Strategies

Several supportive therapeutic approaches are being explored to manage T2D-related pathological mechanisms leading to PDAC. These include natural products (NPs), consisting of bioactive compounds which have shown the potential for the prevention and treatment of many diseases [25] and provide a framework for drug development [26,27]. More than 10,000 phytochemical constituents derived from various plants, valued for their bioactive properties, were suggested as supportive therapeutics for cancer patients, due to their efficacy and low toxicity [28,29]. NPs, in combination with conventional chemotherapeutic agents and radiotherapy as the supportive treatment strategy, may enhance anticancer activity and reduce adverse effects [30]. The anticancer activity of NPs includes reducing the levels of TNF-α, inflammatory cytokines, COX-2, cyclin D1, downregulation of NF-κB, suppression of Bcl-2, an anti-apoptotic protein, and activation of Bax, a pro-apoptotic protein, activation of caspase-3,9, and downregulation of PI3k-Akt and mTOR pathways. Some NPs have shown hypolipidemic and hypoglycemic properties, anti-inflammatory, antioxidant, immunomodulatory, anti-angiogenic, anticoagulant activity, or cardiovascular-protecting properties [28,29]. Such promising activity has been suggested for curcumin from Curcuma longa L., Nigella sativa L. or its constituent thymoquinone, Glycine max (L.) or genistein, Ginkgo biloba L., Gymnema sylvestre (R. Br.) or Momordica charantia L. Several adaptogenic medicinal plants have also been proposed for their ability to protect cells during stress-induced changes in the tissue environment, helping them to resist stress and having a homeostatic effect on various body systems, including the immune, nervous and endocrine systems [31,32,33]. In addition, curcumin, *Nigella sativa* L. and thymoquinone, and Ginkgo biloba L. have shown neuroprotective and cognitive-enhancing properties and partial capacity for restoring the sympathovagal balance and monoamine levels [34,35,36]. 

Another tested approach is nutritional ketosis as the therapeutic strategy for the metabolic management of cancer. Physiological ketosis, achieved by a restricted low-carbohydrate ketogenic diet (LCKD), is aimed at the induction of an energy crisis and glucose deprivation in hyperglycemic and hyperinsulinemia-stressed tissues [37]. Several clinical trials have shown the efficacy of LCKD in different cancer types, particularly in glioblastoma, colorectal, breast, head and neck, and lung cancer [38]. Ketosis, a metabolic state, acts on multiple levels. It targets the Warburg effect, a modified metabolism in cancer cells which uses glycolysis, rather than oxidative phosphorylation, to produce ATP like normal cells. Cancer cells are unable to metabolize ketone bodies (KB) due to the mitochondrial altered morphology and dysfunction. LCKD can decrease glucose levels, deprive cancer cells of energy and create metabolic stress, while normal cells adapt and utilize KB for energy production and survival [39]. Nutritional ketosis reduces insulin and IGF-1 and thus downregulates the cancer cells’ mitogenic activity, the proliferation and generation of inflammatory molecules, increases DNA repair mechanisms, autophagy and mitophagy, telomerase length, inhibits NF-kB, promotes apoptosis, and prevents tumorigenesis [40]. LCKD has been shown to significantly improve glycemic control and reverse T2D [41,42,43,44]. Additionally, signaling of β-hydroxybutyrate (BHB; commonly referred to as ketone) has neuroprotective effects, exhibits tumor-suppressing activity, and has an anti-inflammatory effect via downregulation of inflammatory molecules, such as TNF-α, IL-1,6,18, and prostaglandins. Calorie-restricted nutritional ketosis has shown an anti-angiogenic activity via reduction of HIF-1 and VEGF-receptor expression [38]. LCKD has also shown sympathovagal balance-modulating activity in obese individuals by reducing sympathetic activity (LF: low frequency parameter of heart rate variability (HRV)) and corticosteroid concentration, and increasing parasympathetic cardiac activation (mean RR interval and HF: high frequency parameter of HRV), thus promoting a higher HRV and positive affect on hypothalamic-pituitary-adrenal (HPA) axis and antioxidant capacity [45]. The increased sympathetic and decreased vagal nerve activity can be potentiated by stress and determined by various HRV parameters [46,47]. A lower HRV is often prevalent in cancer patients. Patients with higher HRV and coping mechanisms for stress have shown better adaptability [48,49] and more favorable prognosis, which has also been demonstrated in PDAC patients [50]. 

Limited meta-analyses or reviews have investigated the effect of nutritional or vitamin supplements and plants on PDAC patients. The favorable clinical outcomes and reduced PDAC risk were achieved in combination with chemotherapeutic agents [33,51,52]. The effect of nutraceuticals against PDAC is mediated via a number of mechanisms. The risk of PDAC development can be diminished by early prevention and the treatment of diabetic complications of impaired GT and insulin secretion, IR, or obesity [53]. However, the evidence investigating these common mechanisms with activities of nutraceuticals jointly in both PDAC and T2D is missing. Therefore, the aim of the study is to review available data on the treatment efficacy of NPs and LCKD in T2D and PDAC patients and discuss their possible integration in cancer management. 

## 2. Methods

The literature search included collecting the available information on medicinal plants or phytochemicals used in conjunction with conventional therapies for the treatment of T2D and PDAC. Published original clinical studies for the NPs used in both diseases were identified by searching PubMed, Embase, and Cochrane Library databases. A similar search strategy was conducted for clinical studies of LCKD. Search terms were (pancreatic cancer OR pancreatic ductal adenocarcinoma) AND (type 2 diabetes) AND (curcuma longa OR curcumin) AND (nigella sativa OR black cumin OR thymoquinone) AND (glycine max OR genistein OR soy isoflavone) AND (ginkgo biloba) AND (low-carbohydrate ketogenic diet OR nutritional ketosis). Data were collected from 1998 until August 2021. Clinical studies were selected based on title and abstract first, and further following the selection criteria. The inclusion criteria included original clinical studies (clinical trial phase I–IV, (non)randomized (un)controlled clinical trial, observational or interventional study); NP/LCKD as a primary source of intervention, including the combination of nutraceuticals with chemo-/radiotherapy or current anti-diabetic medication; participants diagnosed with PDAC or T2D; and full-text articles in English. Studies were included only if LCKD and particular NPs were used as monotherapy, not combined with other NPs, and indicated in both PDAC and T2D. Given the scarcity of available data on NPs and LCKD for both conditions, a broader spectrum of clinical studies was acceptable, including one case series clinical study. Exclusion criteria were: studies in vitro or in vivo, reviews, meta-analyses; NP/LCKD in combination with other nutraceuticals; participants diagnosed with other types of cancer or primary disease; and non-full text and non-English language articles. Data collection, including the searching and analysis of clinical studies, was independently performed by two authors. Data were extracted using a self-designed framework, including the study design and duration, sample size, participants characteristics, intervention used, outcome measures, results, and adverse events. The methodological quality of included studies was assessed using a modified Jadad scale, evaluating the randomization, the blinding method, the description of withdrawals and dropouts, the inclusion/exclusion criteria, the description of the method used to assess adverse effects, and the statistical analysis. The scoring system ranged between 0 (0–3, indicating lower quality) and 8 (4–8, indicating higher quality) [54]. The study was conducted according to PRISMA guidelines [55]. 

## 3. Results

**Figure 2:**
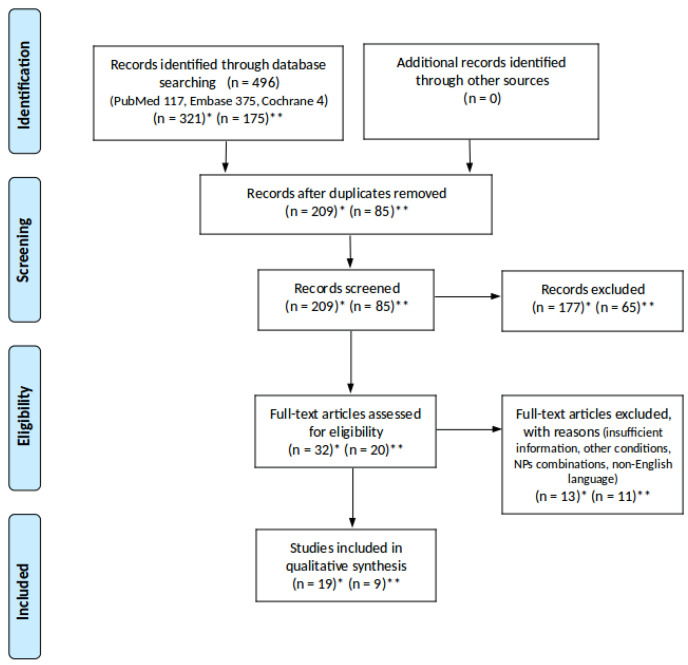
Flow diagram showing the process of clinical trials selection. * Records identified for natural products used in PDAC and T2D; ** records identified for low-carbohydrate ketogenic diet used in PDAC and T2D.

The selection process is presented in the flow diagram (Figure 2), and extracted data are provided in Table 1 and Table 2. The electronic search delivered 321 studies for NPs and 175 studies for LCKD used in PDAC and T2D management. After removing duplicates and screening titles and abstracts, 52 articles remained for full-text assessment. Twenty-four studies were excluded due to insufficient information, focusing on conditions associated with T2D and PDAC, or using a combination of nutraceutical products. The studies using a combination of curcumin products were accepted, due to their low bioavailability and rapid elimination from the body when used as a single agent [56]. Co-administration with piperine, phospholipids, or turmeric oil may increase curcumin absorption in the gut 20-fold and bring beneficial results during treatment [57,58,59]. The studies using micronutrients and other lifestyle recommendations alongside LCKD were also included. Nineteen studies were selected for review, among which eight studies, including 177 participants, used NPs for the management of PDAC [60,61,62,63,64,65,66,67] and 11 studies, including 617 participants, for T2D [68,69,70,71,72,73,74,75,76,77,78] (Table 1). Nine studies using LCKD were included, among which four studies were conducted on 27 patients with PDAC [79,80,81,82] and five studies on 422 diabetic patients [83,84,85,86,87] (Table 2). Aside from studies conducted on patients with T2D, one study [76] with pre-diabetic patients was also included. All 28 studies were peer-reviewed.

### 3.1. Participants’ and Studies’ Characteristics

The participants in all PDAC clinical studies were diagnosed with locally advanced or metastatic disease (or just specified as carcinoma). Participants’ previous therapy varied, including surgery, radiotherapy, chemotherapy, immunotherapy, or none of the prior treatment was specified. Gemcitabine was the most frequent chemotherapeutic used. Other agents were nab-paclitaxel, S-1, FOLFIRINOX, erlotinib, dexamethasone or metoclopramide. The majority of studies (83%) conducted on PDAC patients were open-label, non-randomized uncontrolled interventional phase I or II clinical trials, except for two studies, among which one was a prospective controlled study [79] and one was conducted as a case series study [82]. They lasted from 1 week to 23 months.

Prior medication of participants with T2D was predominantly metformin, then sulfonylureas, a thiazolidinedione, insulin, glucagon-like-peptide-1 receptor agonists, dipeptidyl peptidase 4 and sodium-glucose cotransporter-2 inhibitors, statins, antihypertensives, or no prior treatment. Studies with diabetic patients included open-label non-randomized (12.5%), randomized (25%) controlled clinical trials and randomized double-blind (or participants-blind, n = 1) placebo-controlled clinical trials (62.5%), which are less prone to bias than other designs. The duration of these studies ranged from 10 days to 1 year. Except for two studies [63,78], all clinical trials were single-centered.

### 3.2. Interventions

Active constituents of parental plants were used in all studies, except three studies with *Nigella sativa*, where the plant seeds were processed to a final product [72,73,74], and two studies with *Glycine max*, whose leaves were used without specified constituents [75,76]. The active constituents of *Curcuma longa* were curcuminoids (curcumin, desmethoxycurcumin, bisdesmethoxycurcumin). Curcuminoids were used alone or combined with piperine or turmeric oil or prepared as nano-micelle or phytosome (Meriva^®^) to enhance bioavailability. The other active compound used was thymoquinone from *Nigella sativa* [64]. Two studies used genistein [65], a constituent of *Glycine max*, and the combination of genistein, daidzin, glycitin, a Novasoy^®^ product [66]. *Ginkgo biloba* standardized extract EGb761 (ginkgo flavone glycosides, terpene lactones (ginkgolides, bilobalide) was used in all three studies [67,77,78]. 

All NPs were administered orally in the form of capsules, except for one study, in which 350 mg of *Ginkgo biloba* extract was administered intravenously in 250 mL physiologic saline solution as an infusion [69]. In one study, *Glycine max* leaf powder was incorporated into biscuits [75]. Dosages varied for each NP. They were 8 g, 2 g and 1.5 g of curcumin/curcuminoids per day. Two studies used a lower daily dose of 80 mg of curcumin in nano-micelle form [69] and 500 mg of curcumin with 5 mg of piperine [70]. The application of a nano-carrier allowed use of a lower dose of curcumin and its delivery with increased bioavailability. *Nigella sativa* was administered as seed oil extract capsules, with a dose of 1 g [72] and 5 g [73], and as seed powder capsules, with an overall dose of 2 g per day [74]. One phase-I study used the thymoquinone constituent of *Nigella sativa* in various escalating dosages for participants with different cancers. A dose of 85 mg and 500 mg per day was used in two patients with PDAC [64]. Genistein, a constituent of *Glycine max*, was used in escalating dosages, from 400 mg to 1600 mg [65], and in a dose of 531 mg (Novasoy^®^) per day [66]. A dose of *Glycine max* leaf powder was 10 g (in biscuits) [75] and 2 g (extracted in 70% ethanol) per day [76]. The oral daily dose of *Ginkgo biloba* was 120 mg [77,78]. 

Six open-label studies on participants with PDAC used the combination intervention, including NP and chemotherapeutic agents. Those include curcumin and gemcitabine [61,62], or S-1 (oral fluoropyrimidine derivative) [63], genistein and gemcitabine [65], or in a combination with erlotinib [66], and *Ginkgo biloba* and 5-FU [67]. 

LCKD intervention was compared to a general, low-glycemic, reduced-calorie, or low-fat diet [79,83,85,86,87]. It was used as monotherapy [80,82,84] or in combination with radiation and chemotherapy (gemcitabine, 5-FU) in one study [81]. LCKD treatment comprised of low carbohydrates, high fat, and moderate protein content. However, ketogenic ratios and instructions varied across studies or were not specified in detail. They included, for instance, 2%, 10% or 12% of carbohydrates; 8%, 25% or 28% of proteins; and 90%, 65% or 59% of fat; 1.05–1.75 (fat): 1 (carbohydrate + protein); 20 g (carbohydrate): 120–140 g (fat): 70 g (proteins) per day; <20 g or <30 g of carbohydrate daily. In two studies, LCKD was combined with a calorie supplementation of a medium chain triglyceride oil and ketogenic formula [82] or multivitamins [87]. Two studies only recommended taking additional nutritional supplements in case of adverse events [83,84] and physical exercise for 30 min at least three times a week [83]. 

### 3.3. Outcome Measures

**Figure 3:**
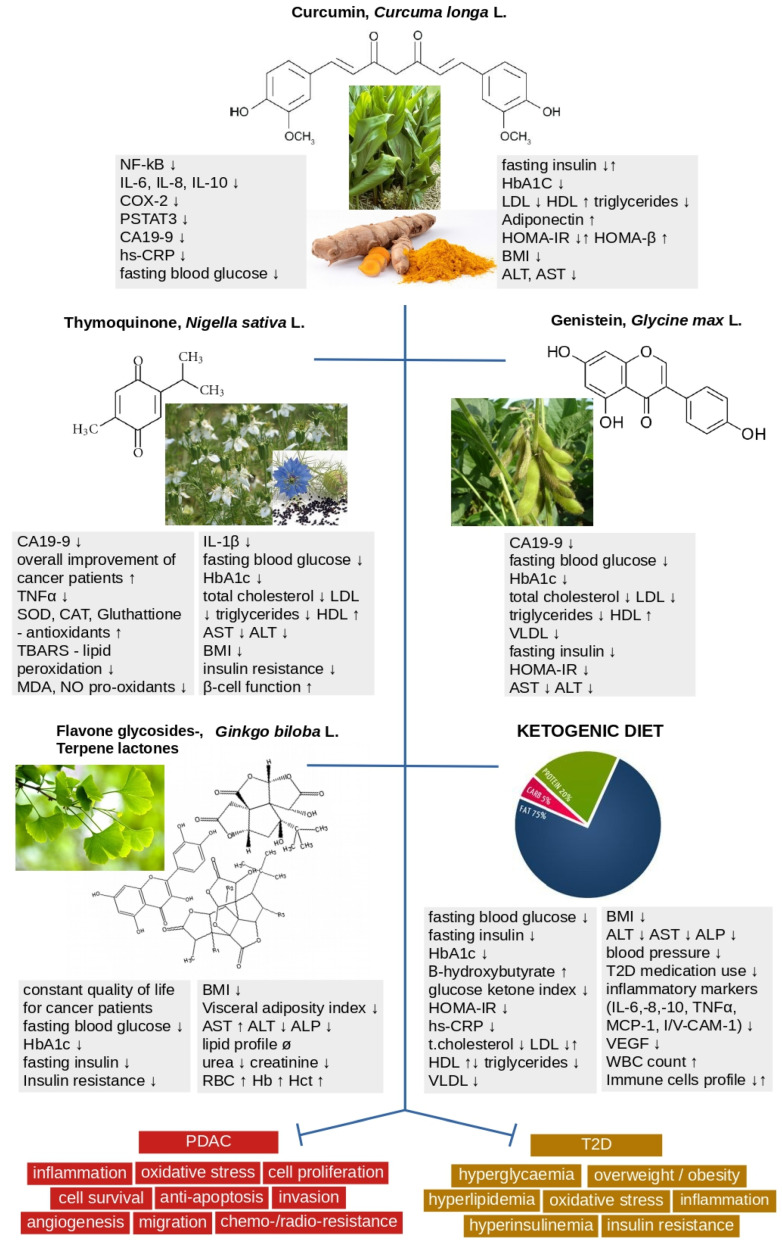
Summary of the markers’ changes in PDAC and T2D patients using nutraceuticals in disease management. NF-κB: nuclear factor kappa B; IL-6,8,10,1β: interleukins 6,8,10,1beta; COX-2: cyclooxygenase-2; PSTAT3: phosphorylated signal transducer and activator of the transcription 3; CA19-9: carbohydrate antigen 19-9; hs-CRP: high-sensitivity C-reactive protein; HbA1C: hemoglobin A1C; LDL: low-density lipoprotein; HDL: high-density lipoprotein; VLDL: very low-density lipoprotein; HOMA-IR: homeostatic model assessment for insulin resistance; HOMA-β: homeostatic model assessment for beta-cell function; BMI: body mass index; ALT: alanine transaminase; AST: aspartate transaminase; ALP: alkaline phosphatase; TNF-α: tumor necrosis factor-alpha; SOD: superoxide dismutases; CAT: catalase; TBARS: thiobarbituric acid reactive substances; MDA: malondialdehyde; NO: nitric oxide; RBC: red blood cells; Hb: hemoglobin; Hct: hematocrit; MCP-1: monocyte chemoattractant protein-1; VCAM-1: vascular cell adhesion molecule 1; VEGF: vascular endothelial growth factor; WBC: white blood cells; ↓/↑ decrease/increase of parameters.

The primary outcome parameters in studies on patients with PDAC differed across studies and for each NP. The common parameters measured were tumor response rate (TRR), overall and progression-free survival (PFS), tumor and inflammation markers, toxicity profile, and quality of life. Additional parameters measured depended on the study’s character and included NF-kB, PSTAT3, liver and renal function, lipid profile or pharmacokinetics, and a maximum tolerated dose. The outcome parameters in studies on patients with T2D and NPs were more consistent, and they included fasting blood glucose, glycated hemoglobin (HbA1c), fasting insulin, IR and β-cell function, lipid profile, BMI, C-reactive protein and adiponectin levels, the function of liver enzymes (AST, ALT), antioxidant capacity and the levels of pro-inflammatory cytokines, measured in two studies [72,74]. Outcome parameters in studies with LCKD were similar to parameters in studies with NPs and diabetic patients. The level of diabetic medication use, inflammatory markers and immune cell profile were additionally measured in two studies [85,87] (Figure 3). Findings also reported the TRR and/or survival in PDAC patients [80,81,82].

Curcumin (*Curcuma longa* L.). Oral curcumin was well tolerated as a monotherapy [60] as well as in combination with gemcitabine [61,62,63]. Only five patients (29%) had to discontinue the treatment for a few days, due to abdominal fullness or pain, and an 8 g daily dose was reduced [61]. In PDAC patients with heterogeneous history of chemotherapy, radiotherapy or surgery, stable disease was achieved in 28–36% of cases [61,62,63], and a partial response was achieved in 9% of cases [61] with a concurrent treatment of curcumin and gemcitabine, achieving a disease control rate of 24% [61] and 61.4% [62]. There was no complete response. However, one patient on curcumin monotherapy experienced a marked tumor regression (73%), with significantly increased cytokine levels and recurrent tumor progression [60]. The median time to progression was 2.5–8.4 months (range 1–12 months) [61,62], and the median overall survival (OS) was 5–10.2 months (range 3.6–24 months) [61,62,63]. Tumor marker CA19–9 decreased in 18% of cases, and 12% of cases maintained normal marker levels [61]. A slow reduction of CA125 was achieved after 1 year in one patient [60]. Elevated levels of cytokines variably changed after treatment with curcumin [60]. However, curcumin significantly reduced the expression of NF-kB, COX-2, and phosphorylated STAT3, which are implicated in tumor-/angiogenesis and growth and are over-expressed in pancreatic and other cancers [60]. Quality of life slightly increased [62]. 

Curcumin achieved more profound changes in patients with T2D. There was a significant decrease in fasting blood glucose [69,71], glycated hemoglobin [69,70], low-density lipoprotein (LDL) cholesterol [69], and serum triglycerides [68]. Insulin levels did not show any significant difference after the treatment with curcumin. There was only a slight decrease in two studies [68,69] and an increase in another study, with slightly increased IR and β-cell function [71]. Total antioxidant capacity, measured in one study, did not show any difference from the baseline [71]. However, curcumin markedly reduced inflammatory C-reactive protein [68,70], increased adiponectin, and anti-inflammatory cytokines [68]. BMI was also significantly decreased in two of four studies [69,70].

Thymoquinone and *Nigella sativa* L. The only study published on this topic including PDAC patients was of poor quality, without outcome measures provided. Findings had a narrative character that included the outcomes for all patients and different types of cancer, including PDAC. The treatment with thymoquinone improved overall general condition and reduction of tumor markers (<25% decrease from baseline) in four of 21 patients. Other parameters, such as lipid profile, renal and liver function, and random blood glucose, did not show any significant changes from baseline [64]. 

The treatment with the *Nigella sativa* product in diabetic patients showed better results. Fasting blood glucose was significantly reduced in all three studies [72,73,74]. Other significant changes in the intervention groups were observed, in the reduction of glycated hemoglobin [73,74], BMI [73] and IR, and in the increase of β-cell function [74]. Despite the slight improvements in lipid profile and liver function, these changes were not significant [73]. *Nigella sativa* supplementation induced an antioxidant activity with more pronounced positive changes after 1 year [74] compared to 8 weeks of intervention [72]. Findings showed a significant elevation in total antioxidant capacity (*p* < 0.002), antioxidant biomarkers—superoxide dismutase (*p* < 0.04), catalase (*p* < 0.003), and glutathione (*p* < 0.03), and a marked reduction in thiobarbituric acid reactive substances (*p* < 0.02) [74], malondialdehyde and nitric oxide, the oxidative stress species [72]. A significant anti-inflammatory effect was not shown, as the TNF-α and interleukin-1β (IL-1β), pro-inflammatory cytokines were reduced only moderately [72]. 

Genistein and *Glycine max* (L.). A combination of genistein with gemcitabine [65] and with gemcitabine and erlotinib [66] resulted in no additional toxicity in PDAC patients. However, there was no survival improvement either. Elevated tumor marker CA19-9 dropped by ≥50% in eight of 16 patients. Quality of life gradually decreased by 10–30% after an average of 12 weeks [65]. Partial response and stable disease were observed in two studies, in 13% and 5%, and 44% and 30% of cases [65,66], respectively. Median PFS was 2.6 months (range 0.7–13.2 months) [65] and 2 months (range 2.0–9.0 months) [66] and median OS was 4.9 months (range 1.5–19.5 months) [65] and 5.2 months (range 4.6-N/A months) [66]. More than a 6-month survival was achieved in 44% [65] or 50% of cases [66], and 1-year survival in 19% of cases [65]. 

*Glycine max* leaf extract intervention revealed a significant reduction in fasting and postprandial blood glucose and glycated hemoglobin in both diabetic [75] and pre-diabetic patients [76]. Another marked improvement in diabetic patients was a lipid profile (total cholesterol, LDL, high-density lipoproteins (HDL), triglycerides) [75], whereas there was no significant difference between pre- and post-treatment shown in pre-diabetic patients. In the latter group, insulin levels and IR were also only moderately decreased, but liver function showed a significant improvement by reducing the levels of transaminases (ALT, AST) [76]. 

*Ginkgo biloba* L. The *Ginkgo biloba* product, in combination with 5-FU, showed a good risk-benefit ratio in the treatment of PDAC, with a low level of adverse events and improvement of the treatment tolerability, with stable quality of life during the treatment period [67]. There was no complete response. Partial response and stable disease were achieved in 9.4% and 21.9% of cases, respectively. The median OS was 5.6 months (range 2.6–7.3 months). More than a 15-month survival was reached by only one patient [67]. 

Administration of *Ginkgo biloba* with metformin resulted in more significant changes [78] than in *Ginkgo biloba* monotherapy intervention [77] in patients with T2D. In the combination treatment, a marked reduction was observed in fasting blood glucose, glycated hemoglobin, serum insulin, BMI, visceral adiposity index, and urea and creatinine levels. *Ginkgo biloba* also significantly contributed to an increase in blood parameters, particularly hematocrit, hemoglobin, and red blood cell count [78]. *Ginkgo biloba* monotherapy studies differed in terms of control groups. There were hyperinsulinemia patients controlled on a diet; those already taking hypoglycemic medication; and patients with pancreatic exhaustion, also taking hypoglycemic medication. The only significant effect was observed during the response to glucose loading in the oral glucose tolerance test. Ingestion of *Ginkgo biloba* caused a marked reduction of plasma insulin in hyperinsulinemic diabetic patients taking hypoglycemic medication. The reducing effect on insulin levels in diet-controlled patients was only minor. In patients with pancreatic exhaustion, ingestion of *Ginkgo biloba* significantly improved β-cell function (increased C-peptide and insulin levels), which, however, did not reduce blood glucose [77]. 

Low-carbohydrate ketogenic diet. LCKD intervention also demonstrated variable outcomes in PDAC patients. In two studies, the patients were a part of a bigger group combining other cancer types [80,82]. Two PDAC patients experienced progressive disease and did not complete the study [80]. Four patients achieved a median OS of 10.7 months. This positive outcome might result from the synergistic effect of LCKD with prior patients’ chemotherapy (gemcitabine or gemcitabine and S-1). Fasting blood glucose was significantly reduced in two of four patients. All four patients showed a marked increase in β-hydroxybutyrate levels and thus a significant reduction of glucose ketone index, which provides information about a state of ketosis and metabolic health [82]. In the study with control to general diet in patients after pancreatectomy, the LCKD group demonstrated significant changes in almost all measured parameters; however, changes between groups were not profound. A reduction of body cell mass was higher in the general diet group, while patients on LCKD had a higher energy intake, better meal compliance, and overall satisfaction. Although urine ketones and C-reactive protein levels increased, this change was insignificant [79]. In the study with concurrent chemo-/radiotherapy, one patient did not complete the LCKD intervention [81]. The patient was on ketosis for only 8 days, but achieved better results in PFS (5.3 months) and OS (10 months) than a patient who completed the study, whose time to progression and OS were 2 months [81]. 

LCKD had a more significant effect on patients with T2D in all five studies [83,84,85,86,87]. The findings revealed a marked decrease in fasting glucose [83,84,85], glycated hemoglobin [83,84,86], fasting insulin [83,84], and IR [84]. The serum lipids measured in four studies were also improved, except in one study, where only triglycerides were significantly reduced [86]. Other pronounced improvements included a reduction in diabetic medication use [83,84], improvement of liver function by decreasing elevated transaminase levels (AST, ALT) [84], and a drop in blood pressure [83]. Inflammation markers and an immune cells profile were measured in two studies [85,87]. LCKD led to a significant reduction in inflammatory C-reactive protein [84,87], IL-8, TNF-α, VEGF, and adhesion molecules E- and P-selectin. Another reduction was achieved in plasminogen activator inhibitor-1, a fibrinolysis inhibitor, monocyte chemoattractant protein-1 [85,87], implicated in the infiltration of monocytes/macrophages at the initiation of inflammation [88] and the monocyte-derived microparticles [85], which are released under inflammatory conditions and are usually elevated in patients with T2D [89]. LCKD did not cause significant changes in immune cell levels in any of the studies [85,87].

### 3.4. Safety Issues

No serious adverse effect was reported for NPs in patients with T2D. Only four patients experienced mild transient nausea after intervention with a *Nigella sativa* product [73]. PDAC patients experienced adverse effects, mostly due to the concurrent chemotherapy, except in one study, where the curcumin dose had to be reduced or discontinued in some patients due to curcumin-specific grade 3 abdominal pain and fullness. However, toxicity related to curcumin did not affect gemcitabine dosing [61]. Hematological or non-hematological toxicity at grades 3–4 appeared in two other studies [62,63], which led to reduction of the curcumin dose or suspension of both gemcitabine and curcumin until recovery. The genistein product did not cause any adverse effects during product monotherapy, nor increased toxicity in combination with gemcitabine [65] or erlotinib [66]. Nevertheless, patients did experience grade 3–4 adverse effects specific to both chemotherapeutics. Similarly, the side effects in the treatment with *Ginkgo biloba* product were related to 5-FU, disease progression, or other medication [67]. 

More adverse events in patients with PDAC were related to chemotherapy or disease progression. LCKD-specific adverse effects included weight loss (73% of cases), hyperuricemia (64%) and, to a minor extent, hyperlipidemia, pedal oedema, anemia, halitosis, pruritus, hypoglycemia, hyperkaliemia, hypokalemia, hypomagnesaemia, and flu-like symptoms or fatigue [80]. Reduced compliance with LCKD intake during concurrent chemo-/radiotherapy was due to adverse effects of the overall treatment regimen [81]. One study reported grade 1–2 adverse effects related to LCKD; however, these were also experienced by patients with other cancer types, and the side effects exclusive to PDAC patients were not specified [82]. The frequency of meal intake-related adverse effects (anorexia, nausea and vomiting, constipation, diarrhea, abdominal pain) was lower in patients after pancreatectomy and on a LCKD than in patients on a general diet, which may suggest LCKD as a better adjuvant complementary treatment strategy [79].

Information about LCKD-related adverse events was not provided in two studies in patients with T2D [85,87]. One study reported problems with headaches, constipation, diarrhea, insomnia, or back pains [83], and another study reported issues with increased constipation [86]. On the contrary, in the latter study, patients with LCKD reported a decrease in headaches, bloating and gas compared to the control group [86]. Adverse effects in diabetic patients differed between two study groups: the continuous care intervention unit and the standard care unit. In the first group, patients experienced fewer side effects, particularly the increased blood urea nitrogen, possibly due to higher protein intake, even though this is not recommended, and two patients experienced subclinical hypothyroidism. In the second group, more serious complications were experienced, including percutaneous coronary intervention (PCI) to left anterior descending stenosis, PCI to the right coronary artery, carotid artery disease, multifactorial encephalopathy, and diabetic ketoacidosis with pulmonary emboli. These adverse effects were not attributed to the intervention [84]. 

## 4. Discussion

Despite partial improvement in several biological parameters, treatment tolerability and stable wellbeing, the studied substances did not significantly improve the treatment response of PDAC patients. In contrast, it is apparent that T2D patients may benefit from the treatment with NPs and LCKD. However, numerous questions remain open, mainly regarding the reliability of reviewed compounds before their integration into clinical practice. 

### 4.1. Mechanisms of Action

Curcuminoids are negative regulators of a transcription factor NF-κB, phosphorylation of STAT3 and an elevated COX-2, which are mediatory contributors to inflammatory processes through the activation of inflammatory cytokine cascade [60,90]. Such inhibition further decreases the expression of proteins implicated in cell proliferation and apoptosis, such as cyclin D1 or c-myc and Bcl-2 or survivin, respectively [91]. The positive effect of curcumin in in vitro and in vivo experiments has also been associated with the downregulation of EGFR, Notch-1 signaling pathways and p-Erk1/2 expressions, which are implicated in PDAC cell growth [92,93]. Curcumin-induced downregulation of elevated prostaglandins E2, TNF-α, interleukin-6,-8,-10, malondialdehyde free radicals and an increase of glutathione and other antioxidant molecules are supported by the additional clinical evidence [59]. However, studied clinical trials did not confirm the beneficial effect of curcumin on PDAC patients’ outcomes. 

Chronic inflammation is associated with hyperglycemia, obesity, metabolic syndrome or IR, and curcumin’s anti-diabetic activity is partially linked to reducing inflammation [94]. Curcumin suppresses NF-kB activity, macrophage infiltration of adipose tissue and, consequently, the expression of C-reactive protein, and increases adiponectin production, which is involved in blood glucose regulation and fatty acid catabolism. Downregulation of serum-free fatty acids by increased oxidation and its utilization in tissues has a hypoglycemic effect, which makes curcumin an ameliorating agent of T2D [94]. Curcumin reduces inflammation by regulating arachidonic acid metabolism, leading to COX, LOX, and nitric oxide synthases suppression [68]. By reducing inflammatory processes, curcumin improves insulin signaling and prevents the progression of T2D [94]. Inflammation and oxidative stress promote glucose and lipid toxicity and deteriorate β-cells’ function [95,96]. Curcuminoids are strong antioxidants and accomplish protective activity by free radical scavenging, performed predominantly by phenolic hydroxyl groups, and by reducing nitric oxide levels, which drives the reactive metabolites [97]. As shown in the reviewed studies, curcumin is capable of decreasing serum cholesterol via suppression of its absorption. The mechanism behind curcumin hypolipidemic activity is suggested by inhibiting the sterol regulatory element-binding transcription factor 1 and fatty acid synthase and by increasing β-oxidation and metabolism of fatty acids, which might prevent a rise of serum lipids [68].

Importantly, curcumin has the potential to act as an epigenetic modulator via inhibition of DNA methyl transferases (DNMTs) and regulation of histone acetyltransferases (HATs) and histone deacetylases (HDACs). Epigenetic inhibitors, such as curcumin, were shown to have the ability to reverse aberrant epigenetic modification and regulate gene expression [98]. Moreover, combined with other anticancer therapies, they could play an essential role in reversing acquired therapy resistance in solid tumors [99]. By regulating the balance of DNA methylation and histone modifications, curcumin and its analogues were shown to reverse T2D complications via modulation of inflammation, over-deposition of extracellular matrix and fibrosis [100].

Thymoquinone is the predominant biologically active essential oil constituent of *Nigella sativa* [101], showing insufficient activity in the reviewed study in patients with PDAC [64]. The reviewed studies revealed the anti-inflammatory and antioxidant activity of *Nigella sativa,* which can benefit both PDAC and diabetic patients. The activity is associated with decreasing the activity of NF-kB and HDACs, synthesis of monocyte chemoattractant protein-1, TNF-α, IL-1β, IL-8, COX-2 and prostaglandin-E2, expression of COX-1 and nitric oxide, increasing superoxide dismutase, catalase, glutathione antioxidants, and decreasing thiobarbituric acid reactive substances and malondialdehyde, the oxidative stress molecules [72,74,102,103]. The antihyperglycemic effect of *Nigella sativa* demonstrated by the reviewed studies might result from the improvement of the β-cell structure and the activity of carbohydrate metabolism enzymes. This mechanism increased insulin levels and decreased oxidative stress on pancreatic β-cells, thereby protecting their integrity and function [104], as well as reducing blood glucose and glycated hemoglobin levels in vivo [102]. 

Similarly to curcumin, genistein also affects tumorigenesis through epigenetic regulations [105]. Besides its effect on DNA methylation, it shows the ability to alter chromatin configuration. In vivo and in vitro studies demonstrated genistein’s ability to inhibit the activation of NF-kB and Akt signaling pathways involved in growth, angiogenesis, cell death, and chemoresistance [106]. NF-kB is unintentionally activated by chemotherapeutic agents, which may explain the chemoresistance. In docetaxel and cisplatin treatment, NF-kB activation was eliminated in the cells with genistein pre-treatment [107]. Another study showed that genistein pre-treatment, followed by gemcitabine chemotherapy, inhibited tumor cell growth by 60–80%, compared to 25–30% when gemcitabine was used alone [108]. The administration of *Glycine max* leaf extract significantly reduced fasting blood glucose, glycated hemoglobin and triglyceride levels and an improved cholesterol profile in diabetic patients in the reviewed studies [75,76]. A high plasma concentration of genistein resulted in a lower risk of T2D or metabolic syndrome complications [109,110,111,112]. Genistein activity in glycemic control may be associated with an increase of glucokinase, an enzyme that phosphorylates glucose to glucose-6-phosphate, hence decreasing glucose-6-phosphate levels [113]. Another mechanism relates to genistein’s binding ability to peroxisome proliferator-activated receptors (PPARs), which affects insulin activity and glucose metabolism [114]. Glucose clearance is regulated by estrogen, particularly insulin pathways-associated proteins, which increase the levels and translocation of GLUT4, the main glucose transporter [115]. Phytoestrogen genistein is able to increase glucose uptake through activation of AMP-activated protein kinase and GLUT4 translocation, thus providing an antihyperglycemic effect [116]. Hyperglycemia contributes to IR in adipose tissue, which results in the hydrolysis of triglycerides to free fatty acids and their release into the liver and blood circulation. Therefore, reduction of serum triglycerides might be associated with genistein hypoglycemic activity, as well as genistein’s ability to increase fatty acid catabolism in the liver [109,117]. In addition, genistein administration showed a pronounced reduction of malondialdehyde levels and an increase in total antioxidant capacity in patients with T2D. It was enhanced through ROS scavenging ability and increased gene expression of antioxidant enzymes [109,118]. 

While the response rate of 5-FU monotherapy in PDAC patients was only 0–10%, gemcitabine alone or in combination therapy with other chemotherapeutics produced an additional response of 5.4–23%, which, however, brought additional toxicity [119]. The combination of *Ginkgo biloba* and 5-FU contributed to a 9.4% TRR, which is comparable with the abovementioned response rates. Co-treatment with *Ginkgo biloba* improved the treatment tolerability and overall quality of life [67]. Complete response in PDAC is scarce and does not influence the patients’ survival rate. However, two cases with a non-resectable secondary and resected locally recurrent PDAC who experienced a complete response after the treatment with gemcitabine and the combination of 5-FU and *Ginkgo biloba* extract (350 mg daily dose intravenously), respectively, were reported. The patient who underwent combination therapy did not experience any severe therapy-associated adverse effects for 10 months since the diagnosis [120]. These favorable effects of *Ginkgo biloba* on PDAC cells have been shown in previous preclinical studies. Kaempferol, *Ginkgo biloba* flavonoid lowered PDAC cell number and inhibited cell proliferation by 70–90%. Decreased proliferation and activated cancer cell death were associated with the reduction of mitochondrial enzyme activity and an increase in apoptotic bodies. By this mechanism and in combination with 5-FU, Kaempferol can sensitize PDAC cells to chemotherapy and provide an additive action of the abrogation of cancer cell proliferation [121]. Similarly, ginkgolic acid, a phenolic compound of *Ginkgo biloba*, decreased the viability of PDAC cells and promoted their apoptosis in vitro and in vivo. It inhibited the tumor growth by decreasing proliferating cell nuclear antigen (PCNA) and abrogated the de novo lipogenesis of cancer cells by the initiation of AMP-activated protein kinase (AMPK) signaling and reduction of the lipogenesis enzymes levels (acetyl-CoA carboxylase, fatty acid synthase) [122]. AMPK, a serine/threonine protein kinase complex, is involved in cellular energy metabolism control and lipid and glucose metabolism regulation [123]. PDAC has been shown to have an increased rate of fatty acid synthesis, which is required for cancer development and survival [124]. 

The Ginkgo biloba effect on lipid and glucose metabolism in patients with T2D was demonstrated in one of two reviewed studies, showing a significant decrease in fasting serum glucose and insulin levels, IR, glycated hemoglobin, visceral adiposity, or BMI [78]. The mechanism of this effect is suggested to be associated with the improvement of β-cell function (increased insulin and C-peptide during increased glucose levels), transferring blood glucose to peripheral tissue while increasing insulin sensitivity and decreasing IR, as well as stimulating lipolytic enzymes [78,125,126]. However, in another reviewed study, 3-month Ginkgo biloba administration significantly improved pancreatic β-cell function only in diabetic patients with pancreatic exhaustion during a glucose loading [77]. In addition, Ginkgo biloba maintained euglycemia in both pre-diabetic and diabetic patients while decreasing the accumulation of platelet-free radicals, which makes ginkgo a potential platelet-activating factor antagonist and free radical scavenger [127]. Free radicals caused by hyperglycemia are implicated in LDL oxidation-induced atherosclerosis development and the impairment of platelet function, which contributes to micro- and macro-vascular complications. Therefore, administration of Ginkgo biloba could prevent such adverse effects in both diabetic and PDAC patients [127,128]. 

LCKD therapy demonstrated significant improvements in lipid profile, fasting serum glucose and insulin levels, liver function, metabolic health, C-reactive protein levels and some inflammatory markers and medication use, predominantly in patients with T2D [83,84,85,86,87]. Except for marked improvements in fasting glucose, β-hydroxybutyrate levels and lipid profile in four advanced PDAC patients and six patients after pancreatectomy [79,82], variable outcomes of LCKD therapy were achieved in patients with different types of cancer [80,82], including partial and a complete response in 19% and 8% of the patients [82]. 

Previous studies have shown that being in ketosis and producing β-hydroxybutyrate (one of the main ketone bodies) can improve glycemic control and reverse T2D and being overweight or obese. It can prevent and halt cancer progression, improve mild cognitive impairment and cardiovascular disease risk factors, such as atherogenic dyslipidemia and inflammation [129,130]. β-hydroxybutyrate production is inversely dependent on insulin levels, as hyperinsulinemia inhibits the rate of ketogenesis, and ketone bodies are cleared through their increased metabolism. On the contrary, low insulin concentration increases ketogenesis and ketone body levels [131]. Low carbohydrate composition of the LCKD and induced nutritional ketosis do not stimulate the pancreas to secrete insulin, resulting in a reduction of blood glucose, glycated hemoglobin and impaired oxygen saturation capacity, hyperglycemia-inhibited fibrinolysis and accumulation of clotting factors and inflammatory signaling [40]. Elevated insulin levels mediate the accelerated cell division through pro-inflammatory signaling molecules, including cytokines, chemokines, or growth factors, leading to prolonged, chronic inflammation, which is one of the mechanisms of neoplastic progression in PDAC [132]. Hyperinsulinemia leads to increased ROS, which further increases the production of inflammatory cytokines, including TNF*-*α, monocyte chemoattractant protein-1, interleukins, or prostaglandins. Under these conditions, mitochondrial DNA is more susceptible to mutations, and malfunctioned mitochondrial electron transport chain becomes the major producer of ROS in cancer cells [133,134]. Elevated intracellular levels of free radicals (O_2_^−•^, H_2_O_2_) in cancer cell mitochondria might be a target for LCKD therapy, involving a mechanism combating oxidative stress, as the state of nutritional ketosis increases the endogenous production of antioxidants, such as glutathione peroxidase, superoxide dismutase, or catalase [135]. Furthermore, the endogenous production of BHB also leads to an increase in intracellular concentrations of nicotinamide adenine dinucleotide NAD^+^, which is vital for NAD^+^—sirtuin activity. NAD^+^ sirtuins connection is responsible for autophagy, mitophagy, and longevity, driving cellular processes, including insulin action and sensitivity, pancreatic β-cells’ function, energy expenditure, mitochondrial and cognitive function, or inflammatory reactions [136,137,138]. 

Most research studies examining the side effects of LCKD have been performed on patients with epilepsy or those aspiring to lose weight [40]. The successful results of these studies suggested LCKD as adjuvant therapy in cancer treatment [139]. Despite no serious adverse effects experienced with LCKD, there are possible risks which might be potentiated by PDAC or T2D. The most common acute side effect is gastrointestinal discomfort, including nausea and vomiting caused by higher fat intake [40]. An appropriate mineral supplementation can prevent the possible risk of a trace minerals deficiency [140]. As a long-term side effect, increased LDL cholesterol, kidney stones, and renal impairment might be experienced by diabetic patients [40], due to the increased elimination of nitrogenous waste products from protein metabolism. Although long-term daily protein intake and the related adaptive response of renal function did not show adverse effects in healthy individuals, these dietary changes might have an impact on kidney function in patients with T2D or PDAC, who are more susceptible than others [141]. 

### 4.2. Intervention’s Quality and Safety

The popularity and consumption of NPs have increased worldwide, driving more research on their quality and safety profiles [142]. Both medicinal plants and single compounds were used in combination in the reviewed studies. Medicinal plants contain numerous compounds, whose complex interactions may potentially provide a powerful clinical effect. However, these interactions are difficult to and usually not thoroughly examined. The pharmaceutical model using a single active compound can fairly easily explain the mechanism behind a compound’s activity and how can it be exploited for drug manufacturing; however, this could result in loss of beneficial multi-constituent mixtures of the whole plant [143]. Using the single compound or plant extract is also confounded by factors specific to each patient, including dosage and use of other medication, type and stage of disease, and medication- or disease-induced adverse effects or age [143]. In addition, for single compounds, semi-synthetic variants, co-administration with other compounds or in different formulations could often compensate for poor pharmacokinetic properties (poor absorption, fast metabolism, and elimination) and bioavailability of the parental compound [144]. Moreover, -omics technologies, particularly metabolomics and proteomics, can contribute to the standardization of plant extracts and determine the specific phytochemicals that can reduce adverse effects caused by pharmaceutical drugs or make drugs more efficient [145]. 

### 4.3. Clinical Perspectives

As shown by reviewed studies, the pharmacological synergy of standard chemotherapies combined with NPs may provide therapeutic advantages through phytochemical complexity and multiple constituents of herb-herb interactions [143]. The efficacy of the poly-NPs formula has already been demonstrated in clinical practice [146] within the management of some cancer types (such as colorectal, breast and prostate cancers, cervical neoplasia, Barrett’s metaplasia, and other gastrointestinal malignancies) and T2D [29,147,148,149,150]. However, in evaluated clinical studies, the results were more favorable for diabetics than for PDAC patients. PDAC development could potentially be prevented by early intervention at the onset of diabetic symptoms, particularly in type 3c diabetes, in which further prompt examinations can detect a potentially curable tumor [8,53]. Since diabetes is linked to PDAC, distinguishing between T2D and type 3c, and implementing pharmacological and non-pharmacological interventions might be a good preventative strategy [151]. 

The treatment combining LCKD, gemcitabine or FOLFIRINOX, hyperthermia and hyperbaric oxygen therapy in patients with metastatic PDAC achieved longer survival outcomes (median OS 15.8 (10.5–21.1) months) and PFS of 12.9 (11.2–14.6) months [152]. This approach targets impaired mitochondrial energy mechanisms in mutated cancer cells, which is glucose-dependent. LCKD, 12-hour fasting and insulin administration prior to chemotherapy enhanced the effect of chemotherapeutics by making the membranes more permeable, depriving cancer cells of glucose and developing metabolic oxidative stress on the cells [153,154]. Hyperthermia sensitizes cancer cells to radiotherapy and chemotherapy, and hyperbaric oxygen delivery under high pressure can resolve the problem of hypoxia in cancer cells [155,156]. A combination with curcumin could further radiosensitize pancreatic tumor cells and bring additional treatment benefits [157].

Enteral and parenteral LCKD could be offered as an option to cancer patients in hospitals. These patients usually receive higher glucose-containing feeds, leading to hyperglycemia that increases systemic inflammation, which might contribute to cancer progression or the increased incidence of infection [158,159]. Moreover, hyperglycemia-induced elevation of insulin might increase the activity of the sympathoadrenal system, which is known for its cancer-stimulating effect [160]. Low-carbohydrate, high monounsaturated fatty acids tube feeding has been associated with a significant improvement in glycemic control (HbA1c), fasting and postprandial blood glucose in diabetic patients who were also taking antidiabetic medication [161,162,163].

The combination of LCKD, silybin from *Silybum marianum* plant and omega-3 polyunsaturated fatty acids has been found to be a good nutritional strategy to prevent cachexia [164]. This is performed by reducing tumor growth and inflammatory cytokine secretion (IL-6,-8, TNF-α), activating pro-apoptotic molecules, reducing glycolysis proteins, regulating impaired metabolism and immune responses, or preserving skeletal muscle mass. However, decisions regarding the application of LCKD for advanced PDAC cachexic patients are still inconclusive [164,165]. 

The stimulating effect of nerves innervating cancer tissue on tumor growth and invasiveness results in augmentation of peripheral stress-inflammatory responses that give feedback to the CNS, thus affecting patients’ mental state (e.g. emotional tension, depression, impaired cognition, sleep disturbances) and facilitating a vicious cycle of further stress responses [166]. Since adrenergic and inflammatory responses may work in a synergistic and mutually enhancing manner, in addition to combination therapy of NPs and LCKD, and their immune-inflammatory modulation activity, both β-blockers and COX-2 inhibitors can be suggested as promising treatment strategies for improving cancer outcomes. The clinical evidence has also shown the advantage of such an approach in the perioperative period, when the inhibition of perioperative stress-related inflammatory responses to surgery may prevent metastasis, eliminate residual disease, and improve survival [24,167]. To reduce perceived stress and physiological responses, curcumin has shown a beneficial effect in the activation of vagal afferent neurons and restoring the sympathovagal balance. It has been reported to possess antidepressant activity by increasing serotonergic and dopaminergic transmission and suppression of monoamine oxidase, ROS formation, and inflammatory signaling. The beneficial effect of curcumin has also been reported in diabetes-induced CNS dysfunction, caused by fluctuations of acetylcholine neurotransmitters levels, resulting in cognitive impairment [34,168,169]. *Nigella sativa* oil administration has increased 5-hydroxytryptamine (5-HT/serotonin) and tryptophan brain and plasma levels, hence offering antidepressant and anxiolytic activity, which has also been shown through the regulation of γ-aminobutyric acid (GABA) and nitric oxide (NO) levels by thymoquinone [35,170]. Administration of *Ginkgo biloba* extract significantly improved mental and physical activity, reduced fatigue and anxiety via regulation of dopamine and serotonin levels, and inflammatory glial-derived proteins, as well as reversing cerebral hypoperfusion by regulation of neuroinflammation and the cholinergic system [171,172].

Similarly to NPS, LCKD also exhibits sympathovagal balance-modulating activity, relating to promoting a higher HRV and antioxidant capacity [45,173]. HRV analysis has shown potential for monitoring physiological and psychological wellbeing, thus giving an opportunity for biofeedback intervention and improvement of survival [174]. HRV biofeedback has demonstrated positive outcomes through learning how to build up resilience against stress by training to achieve optimal performance or HRV coherence. Higher HRV coherence optimizes autonomic-cardio-respiratory homeostasis, which better helps to sustain the energy for recovery processes during and after treatment [175]. Thus, apart from the combination of NPs with standard chemo- and radio-therapy, a NPs-concomitant, stress-reducing or HRV-improving strategy (psychotherapy, HRV biofeedback, β-blocker treatment) may be of interest in the search for new supportive approaches that could bring additional benefit for PDAC patients through the synergy in described NPs effects and improved autonomic balance.

### 4.4. Study Limitation

The major limitation of this study lays in the heterogeneity of the administered treatments, as well as the studies being available only in English. A small sample size in the studies, especially in the ones with PDAC patients, and the short duration of treatment, in some cases for only few weeks, do not provide sufficient dose- or ketosis-response data for NPs or LCKD to be evaluated and implemented as adjuncts to the anticancer medication. More studies with a larger group of participants, of a longer duration, and with synergistic interventions are therefore needed to assess the effect of NPs and LCKD in integrative and supportive anticancer treatment.

## 5. Conclusions

The data obtained from clinical studies demonstrate the ability of NPs and LCKD to affect multiple biological parameters implicated in the pathology of PDAC and T2D. The few clinical studies in which NPs and LCKD were used as monotherapy or in combination with conventional anticancer medications did not improve response and survival in PDAC patients. However, both interventions showed significant efficacy in the treatment of T2D. Therefore, the present study indicates that NPs and LCKD can have a significant association with reduction of the risk of PDAC development and progression. The risk of PDAC development could be prevented by early intervention at the onset of diabetic symptoms, in which further prompt examinations can detect a tumor at a potentially curable stage. There is no population screening program for PDAC, due to its low incidence. Since T2D is linked to PDAC, implementing an early intervention might be a good screening therapeutic strategy. The interventions can further contribute to improvement in biological parameters and treatment tolerability, maintain stable quality of life, and may benefit patients with pancreatic neoplasms. With the discerned safety of NPs and LCKD, and the ongoing acceptability of nutraceuticals and advances in the field, we believe that supportive management of PDAC is warranted and should be supported further by clinical trials. However, larger-scale research studies and new approaches, with more effective combinations of interventions and the optimal therapeutic window, are needed to overcome current limitations and reliably assess the role of NPs and LCKD in PDAC prevention and treatment. Future studies may also consider initiating a debate with clinicians on using evidence-based nutraceuticals within supportive cancer management; encouraging and educating patients within early prevention programs; and designing the therapeutic protocols and practices of supportive cancer management and their implementation in a healthcare system.

## Figures and Tables

**Table 1:** Characteristics of clinical studies of natural products suggested as adjunct therapeutics for pancreatic adenocarcinoma and type 2 diabetes.

Author	Study Design, Duration, Quality	Participants (Sample Size, Diagnosis)	Intervention Preparation Used	Outcome Measures	Results	Adverse Events
*Curcuma longa* L./Curcumin						
Dhillon et al., 2008 [60]	Open-label non-randomized phase II clinical trial 8 weeks, up to 18 months Jadad score: 4	N = 25 Patients with pancreatic adenocarcinoma M = 13; F = 12; Median age: 65 years (range 43–77) Prior therapy: surgery, radiotherapy, gemcitabine -/+ other chemotherapy, erlotinib, no therapy Healthy volunteers N = 48–62 (depending on the cytokine being measured)	Curcumin (1 capsule—1000 mg/1 g of curcuminoids: 900 mg curcumin, 80 mg desmethoxycurcumin, 20 mg bisdesmethoxycurcumin) – Dose: 8 g daily p.o.	Cytokine levels: IL-6,-8,-10, IL-1RA/receptor antagonist Nuclear factor-κB/NF-kB (p65) Cyclo-oxygenase 2/ COX-2 Phosphorylated signal transducer and activator of transcription 3 (PSTAT3)	∙ Patients – Cytokines: elevated levels (IL-6,8,10) at baseline→ variable level changes after the treatment ↓↑ – Mean ± SD baseline and post-treatment (↓↓/↑↑ *p* < 0.05, significant difference): – NF-kB ↓ 74.50 ± 10.00→65.80 ± 14.20 (*p* = 0.131) – COX-2 ↓↓ 60.80 ± 12.53→44.7 ± 17.37 (*p* = 0.029) – PSTAT3 ↓↓ 40.20 ± 15.76→ 21.10 ± 13.30 (*p* = 0.009) – Tumor regression, cytokines levels ↓ in few patients – Stable disease for over 18 months, ↓ tumor lesions: one patient – 73% reduction in tumor for a month, CA125 level ↓: one 1 patient – Stable weight and wellbeing for 8 months, but progression in non-target lesions: one patient ∙ Volunteers IL-6, IL-8, IL-10 → undetectable serum levels IL-1RA→detectable serum levels NF-kB, pSTAT3 → not activated	No serious side effects reported
Epelbaum et al., 2010 [61]	Open-label non-randomized phase II clinical trial 1 week to 12 months Jadad score: 3	N = 17 Patients with pancreatic adenocarcinoma (locally advanced or metastatic) M = 10; F = 7; Median age: 69 years (range 54–78)	Curcumin (1 capsule—500 mg of curcuminoids: curcumin 450 mg, desmethoxycurcumin 40 mg, bisdesmethoxycurcumin 10 mg) – Dose: 4000 mg of curcumin b.i.d. p.o. (overall 8000 mg) + Gemcitabine—1000 mg/m^2^ IV weekly for 3 of 4 weeks (two cycles)	Overall survival (OS) Tumor response rate Clinical benefit rate (CBR) Time to tumor progression (TTP) CA 19-9 serum levels Toxicity profile	– Early discontinuation of the overall treatment due to toxicity: five patients – Sudden death: one patient – CBR: achieved in four patients – Local control/response rate 45.5% (2–12 months): – Partial response rate: one patient for 7 months; – Stable disease: four patients for 2, 3, 6, 12 months, respectively; – ↓ CA19-9: three patients baseline vs. post-treatment: 90,830→63,130, 714→96, 214→42 – CA19-9 normal levels: two patients – TTP—median 2 ½ months (range = 1–12 months): six patients – OS—median 5 months (range = 1–24 months)	– Gastrointestinal toxicity (grade 3): fullness, upper abdominal pain => curcumin dose reduced (two patients) or discontinued (five patients) – Toxicity specific to curcumin did not affect gemcitabine dosing – Hematological toxicity (grade 1): neutropenia, thrombocytopenia (four patients)
Pastorelli et al., 2018 [62]	Single arm prospective phase II clinical trial 9 cycles +, cycle—every 28 days Jadad score: 4	N = 44 Patients with pancreatic adenocarcinoma (locally advanced or metastatic) M = 29; F = 15; Median age: 66 years (range 42–87) Prior treatment: dexamethasone 8 mg or metoclopramide 10 mg, i.v.	Curcumin (Meriva^®^—phytosome/phospholipids complex of curcumin, one capsule—500 mg) – Dose: 2000 mg/day p.o. + Gemcitabine—10 mg/m^2^/min, infused over 100 min. and diluted in 500 mL normal saline—a dose-intense infusion on 1, 8, 15 days; two–14 cycles	Tumor response rate Progression-free survival (PFS) Overall survival (OS) Quality of life assessment Inflammation markers (CRP; sCD40L; cytokines IL-8,-6, MIP-1; adhesion molecules) Full blood counts CA19-9 serum levels Toxicity profile	– Partial response: 27.3% patients – Stable disease: 34.1% patients – Progression disease: 38.6% patients – Disease control rate: 61.4% – Median OS at follow-up time of 26 months: 10.2 months (95% CI, 8.8–11.7) – Median PFS: 8.4 months (95% CI, 5.0–11.8) – Median OS (locally advanced pancreatic cancer): 16 months – Median OS (metastatic disease): 8.5 months – MIP-1α serum levels ↓ – Patients non-responsive to treatment/high baseline levels of IL-6, sCD40L, CRP → poor response, worse OS, ↑ sCD40L after first cycle of chemotherapy – Patients responding to treatment → no significant variations of biomarkers between baseline and first/third cycles of the treatment – A slight increase in quality of life	– Hematological toxicities grade 3–4 due to dose-intense schedule of Gemcitabine – Neutropenia (grade 3–4, 38.6%) – Anemia (grade 3–4, 6.8%) – Thrombocytopenia (grade 3–4, 6.8%) – Fatigue (grade 1–2, 29.5%) – Nausea and vomiting (grade 1–2, 2%) – Oral mucositis (grade 1–2, 6.8%) – Diarrhea (grade 1–2, 9%; grade 3–4 2.2%)
Kanai et al., 2011 [63]	Open-label phase I–II clinical trial (2 centers) >6 months Jadad score: 4	N = 21 Patients with pancreatic adenocarcinoma M = 13; F = 8; Median age: 67 (range 44–79) Prior treatment: surgery, radiotherapy, chemotherapy (Gemcitabine/S-1, Gemcitabine alone)	Curcumin (complex of curcuminoids: curcumin 73%, demethoxycurcumin 22%, bisdemethoxycurcumin 4%) – Dose: 8 g daily p.o. + Gemcitabine/S-1 Combination (19 patients); Gemcitabine monotherapy (two patients) – Dose: 1.000 mg/m^2^ of gemcitabine i.v. on days 1, 8, 60 mg/m^2^ of S-1 p.o. for 14 consecutive days every 3 weeks	phase I: Safety, Treatment completion rate (TCR) phase II: Tumor response rate Overall survival (OS) Compliance rate of curcumin Blood cell count CEA (carcinoembryonic antigen), CA19-9 Toxicity profile Plasma curcumin levels	– TCR: 100% (95% CI 84–100%; *p* < 0.001) – Compliance rate: 90% (95% CI 70–99%) – Median OS: 161 days (95% CI 109–223 days) – 1-year survival rate: 19% (95% CI 4.4–41.4%) – No partial or complete response – Stable disease: 28% based on RECIST – Plasma curcumin levels—from 29 to 91 ng/mL	Hematological toxicity: – Leucopenia (grade 4, 33%) – Neutropenia (grade 3–4, 38%) – Thrombocytopenia (grade 3–4, 10%); – ↓ Hemoglobin (grade 3–4, 19%) – Non-hematological toxicity – Fatigue, drowsiness, anorexia – Obstruction of the GIT, edema – Stomatitis, nausea/vomiting, diarrhea, skin rash, fever due to infection => Reduced dose of curcumin to 6 g/day or chemotherapy and curcumin suspended until recovery
Adibian et al., 2019 [68]	Randomized, double-blind, placebo controlled clinical trial 10 days Jadad score: 7	N = 44 Patients with type 2 diabetes Curcumin group N = 21 M = 13; F = 8; Mean ± SD age 58± Control group N = 23 M = 9; F = 14; Mean ± SD age: 60 ± 7	Curcumin (1 capsule 500 mg of curcuminoids: curcumin 347 mg, demethoxycurcumin 84 mg, bisdemethoxycurcumin 9 mg, turmeric oil 38 mg) – Dose: 500 mg t.d.s. p.o. Placebo (one capsule, 444 mg of rice flour)	Lipid profile (triglyceride) total, HDL, LDL, cholesterol high-sensitivity C-reactive protein (HS-CRP), adiponectin (anti-inflammatory cytokine) Anthropometric parameters: weight, height, waist, hip circumferences, BMI	Mean change ± SD in curcumin group and placebo group, respectively (↓↓/↑↑ *p* < 0.05 in the intervention group, significant difference): – Serum triglycerides ↓↓ (−14.2 ± 30.6 vs. −5.2 ± 36.6) – LDL ↓ (−3.7 ± 21.4 vs. −7.6 ± 34.7) – HDL ↑ (0.3 ± 2.2 vs. 2.8 ± 5.3) – hs-CRP ↓↓ (−2.5 ± 4.3 vs. 0.8 ± 3.2) – Adiponectin ↑↑ (12.1 ± 7.7 vs. 7 ± 7.1) – mean weight ↓ vs. control – Fasting blood glucose ↓ in curcumin group vs. baseline – Insulin levels ↓ vs. control – HBA1c ↓ vs. control – BMI ↓ in curcumin group, ↑ in control group	Not provided
Rahimi et al., 2016 [69]	Randomized double-blind placebo controlled clinical trial 3 months Jadad score: 6	N = 70 Patients with type 2 diabetes Curcumin group N = 35 M = 17; F = 18; Mean ± SD age: 56.34 ± 11.17 Control group N = 35 M = 14; F = 21; Mean ± SD age: 60.95 ± 10.77	Curcumin (Nano-curcumin/SinaCurcumin^®^—80 mg of curcumin in the form of nano-micelle) – Dose: 80 mg/daily p.o. Placebo (N/A)	Fasting blood glucose (FBG) Glycated hemoglobin (HbA1C) Estimated average glucose (eAG) Lipid profile (total cholesterol (TC), HDL, LDL cholesterol, triglyceride (TG) BMI	Mean baseline and post-treatment in Curcumin group and placebo group, respectively (↓↓/↑↑ *p* < 0.05 in the intervention group, significant difference): – FBG (mg/dL) ↓↓ (135.5→120.29 vs. 148.30→176.0) – HbA1C (%) ↓↓ (7.59→7.31 vs. 7.49→9.00) – eAG ↓↓ (171.2→167.00 vs. 168.4→211.6) – TC (mg/dL) ↓ (163.4→158.62 vs. 162.4→149.00) – LDL (mg/dL) ↓↓ (96.57→91.04 vs. 99.78→84.00) – HDL (mg/dL) ↑↑ (54.30→60.95 vs. 60.35→55.00) – TG (mg/dL) ↑↑ (109→131 vs. 142→113) – BMI (kg/m^2^) ↓↓ (26.92→25.57 vs. 27.27→27.50)	Not provided
Panahi et al., 2018 [70]	Randomized double-blind placebo controlled clinical trial 3 months Jadad score: 5	N = 100 Patients with type 2 diabetes Curcumin group N = 50 M = 25; F = 25; Mean ± SD age: 43 ± 8 Control group N = 50 M = 26; F = 24; Mean ± SD age: 41 ± 7	Curcumin (Curcumin C3 Complex ^®^, curcuminoids: curcumin, demethoxycurcumin, bisdemethoxycurcumin + Bioperine ^®^) – Dose: one capsule/500 mg of curcumin, and 5 mg of piperine daily p.o. Placebo (capsule with unknown content with added 5 mg of piperine)	Fasting insulin (FI) Fasting glucose (FG) Glycated hemoglobin (HbA1c) High-sensitivity C-reactive protein (hs-CRP) Aspartate aminotransferase (AST) Alanine aminotransferase (ALT) Homeostatic model assessments of insulin resistance (HOMA-IR) and beta-cell function (HOMA-β) BMI, weight	Mean change ± SD in curcumin and placebo group, respectively (↓↓/↑↑ *p* < 0.05 in the intervention group, significant difference): – FI (mIU/L) ↓ (−0.9 ± 3 vs. −0.7 ± 2) – HbA1c (%) ↓↓ (−0.9 ± 1.1 vs. −0.2 ± 0.5) – HOMA-IR index ↓ (−0.2 ± 0.4 vs. −0.1 ± 0.3) – HOMA-β ↑ (2.7 ± 16.2 vs. −4.4 ± 16.1) – FG (mg/dL) ↓ (−9 ± 16 vs. −3 ± 11) – hs-CRP ↓↓ (−0.6 ± 0.8 vs. 0.02 ± 0.6) – Creatinine (mg/dL) ↓ (−0.2 ± 0.3 vs. −0.1 ± 0.3) – ALT (U/L) ↓ (−2 ± 6 vs. −1 ± 5) – AST (U/L) ↓ (−3 ± 5 vs. −0.3 ± 4) – BMI (kg/m^2^) ↓↓ (−0.5 ± 0.5 vs. 0.2 ± 0.7) – Weight (kg) ↓↓ (−1.4 ± 1 vs. 0.7 ± 2)	No side effects reported
Hodaei et al., 2019 [71]	Randomized double-blind placebo controlled clinical trial 10 weeks Jadad score: 7	N = 44 Patients with type 2 diabetes Curcumin group N = 21 M = 13; F = 8; Mean ± SD age: 58 ± 8 Control group N = 23 M = 9; F = 14; Mean ± SD age: 60 ± 7	Curcumin (1 capsule 440 mg of curcuminoids:347 mg curcumin, 84 mg desmethoxycurcumin, 9 mg bisdesmethoxycurcumin; and 38 mg of turmeric oil) – Dose: 500 mg t.d.s. p.o. (total 1500 mg) Placebo (one capsule 444 mg of cooked rice flour) – Dose 1 capsule t.d.s. p.o.	Fasting blood glucose (FBG) Total antioxidant capacity (TAC) Malondialdehyde (MDA) Fasting insulin (FI) HbA1c HOMA-β HOMA-IR Weight, BMI	Mean change ± SD in curcumin and placebo group, respectively (↓↓/↑↑ *p* < 0.05 in the intervention group, significant difference): – FBG (mg/dL) ↓↓ (−7 ± 2 vs. 3 ± 0.2) – FI (mU/L) ↑ (0.2 ± 3 vs. 1.4 ± 1.3) – HbA1c (%) ↓ (−0.3 ± 0.4 vs. 0.1 ± 0.5) – HOMA-IR ↑ (0.4 ± 21 vs. 12 ± 4) – HOMA-B ↑ (3 ± 21 vs. 12 ± 37) – TAC ↓ (−0.01 ± 0.01 vs. 0.1 ± 0) – MDA (μmol/L) ↑ (0.5 ± 0.1 vs. 0.5 ± 0.1) – Mean weight (kg) ↓↓ (−0.64 ± 0.22 vs. 0.19 ± 0.37) – BMI (kg/m^2^) ↓ (−0.3 ± 0.03 vs. −0.1 ± 0)	No serious side effects reported
*Nigella sativa* L./Thymoquinone						
Al-Amri et al., 2009 [64]	Open-label non-randomized phase I clinical study Median 3.71 weeks (range 1–20 weeks) Jadad score: 2	N = 21 Patients with various types of cancer, including pancreatic adenocarcinoma N = 2 (Others: non-small cell lung carcinoma, prostatic, colonic, gastric, renal cell, hepatocellular carcinoma, leiomyosarcoma, diffuse large B-cells lymphoma M = 11; F = 10; Median age: 56 (range 23–92)	Thymoquinone—dose: 1 mg/kg/day, 6 mg/kg/day, 10 mg/kg/day, p.o. Dose increased up to 2600 mg/day Thymoquinone dose in patients with pancreatic cancer: 85 mg/day, 500 mg/ day	Toxicity profile Complete blood count (CBC) Renal function (RFT) Liver function (LFT) Random blood glucose (RBS) Lipid profile Erythrocyte sedimentation rate Tumor markers (CEA, CA125, CA19-9, CA153, BHCG, AFP, PSA, LDH) Prothrombin time (PT) Partial thromboplastin time (PTT)	– Overall improvement in patient’s general condition: four patients – Increased weight gain of 2 kg: four patients – Reduction of tumor markers, but not by more than 25% of baseline levels (measured values not provided) – CBC, RFT, LFT, RBS, lipid profile, ESR: no significant changes from baseline (measured values not provided)	No side effects reported
Hadi et al., 2018 [72]	Randomized double-blind placebo controlled clinical trial 8 weeks Jadad score: 6	N = 43 Patients with type 2 diabetes *Nigella sativa* group N = 23 M = 10; F = 13; Mean ± SD age: 51.4 ± 9.2 Control group N = 20 M = 10; F = 10; Mean ± SD age: 56 ± 3.4	*Nigella sativa* (one capsule 500 mg of N.sativa oil extract) – Dose: 500 mg b.i.d. p.o. Placebo – Dose: 500 mg capsule b.i.d. p.o. (content of capsule not provided)	Fasting blood glucose (FBG) Pro-inflammatory cytokines: Tumor necrosis factor-α (TNFα) Interleukin 1β (IL-1β) Pro-oxidant biomarkers: Nitric oxide (NO) Malondialdehyde (MDA) Antioxidant biomarkers: Superoxide dismutase (SOD) Catalase (CAT)	Mean change ± SD in *Nigella sativa* and placebo group, respectively (↓↓/↑↑ *p* < 0.05 in the intervention group, significant difference): – FBG (mg/dL) ↓↓ (−23 ± 39.1 vs. 8.5 ± 2.2) – TNFα (pg/mL) ↓ (−1.3 ± 4.2 vs. 0.45 ± 3.4) – IL-1β (pg/mL) ↓ (−0.37 ± 3.4 vs. 1.38 ± 1.9) – SOD (U/mL) ↑↑ (7.5 ± 16.9 vs. −7.1 ± 16.7) – CAT (U/mL) ↑ (1.8 ± 27.6 vs. −0.86 ± 4.2) – MDA (nmol/L) ↓↓ (−0.7 ± 1.3 vs. 0.98 ± 2.6) – NO (nmol/L) ↓ (−0.6 ± 1.5 vs. −0.16 ± 1.6)	No severe side effects reported
Hosseini et al., 2013 [73]	Randomized double-blind placebo controlled clinical trial 3 months Jadad score: 6.5	N = 70 Patients with type 2 diabetes *Nigella sativa* group N = 35 M = 14; F = 21; Mean ± SD age: 48.74 ± 7.33 Control group N = 35 M = 16; F = 19; Mean ± SD age: 50.72 ± 5.69	*Nigella sativa* – Dose: 2.5 mL b.i.d. p.o. after meals (5 mL of oil daily; cold press *N. sativa* oil) Placebo – Dose: 2.5 mL of mineral oil t.d.s. p.o. – 0.1 mL of the mixture of chlorophyl and red chili extract was added to placebo and *N. sativa* oil to achieve similar appearance and flavor	Fasting blood glucose (FBG) 2h-postprandial blood glucose (2hppBG) Glycated hemoglobin (HbA1c) Lipid profile (total cholesterol/TC, LDL, HDL, triglyceride) Aspartate transaminase (AST) Alanine transaminase (ALT) Alkaline phosphatase (ALP) Creatinine levels, BMI	– Mean change in Nigella sativa and placebo group, respectively (↓↓/↑↑ p < 0.05 in the intervention group, significant difference): – FBG (mg/dL) ↓↓ (−10.15 vs. 3.61) – PBG (mg/dL) ↓↓ (−8.25 vs. 1.32) – HbA1c (%) ↓↓ (−3.40 vs. −1.02) – BMI (kg/m2) ↓↓ (−4.18 vs. 0.64) – TC (mg/dL) ↓ (−3.16 vs. 2.75) – Triglyceride (mg/dL) ↓ (−4.43 vs. 6.00) – HDL (mg/dL) ↑ (1.03 vs. 3.90) – LDL (mg/dL) ↓ (−1.8 vs. 2.96) – Creatinine (mg/dL) ↓ (−4.39 vs. 4.34) – AST (U/L) ↓ (−1.45 vs. −3.02) – ALT (U/L) ↓ (−2.92 vs. −1.31) – ALP (IU/L) ↑ (1.80 vs. 3.20)	No serious side effects reported – Mild transient nausea: four patients – No liver enzyme and kidney functional adverse effects observed
Kaatabi et al., 2015 [74]	Participant-blinded placebo controlled clinical trial 1 year Jadad score: 5.5	N = 114 ∙Patients with type 2 diabetes (on standard hypoglycemic medication: sulfonylureas, metformin) *Nigella sativa* group N = 57 M = 33; F = 24; Mean ± SE age: 46.82 ± 1.14 Control group N = 57 M = 30; F = 27; Mean ± SE age: 46.12 ± 0.85	*Nigella sativa* (1 capsule 500 mg of N.sativa seed powder) – Dose: 1 g (two capsules) b.i.d. p.o. (overall 2 g/day) Placebo – Dose: 260 mg of charcoal capsules	Fasting blood glucose (FBG) Glycated hemoglobin (HbA1c) C-peptide Total antioxidant capacity (TAC) Superoxide dismutase (SOD) Catalase (CAT) Glutathione thiobarbituric acid-reactive substances (TBARS) Insulin resistance β-cell function	Mean baseline and 12-month treatment in *Nigella sativa* and placebo group, respectively (↓↓/↑↑ *p* < 0.05 in the intervention group, significant difference): – FBG (mg/dL) ↓↓ (195→172 vs. 180→180) – HbA1c (%) ↓↓ (8.6→8.2 vs. 8.2→8.5) – C-peptide (ng/mL) ↓ (2.9→2.8 vs. 2.9→2.8) – Insulin resistance ↓↓ (3.0→2.5 vs. 2.5→2.5) – Β-cell function (%) ↑↑ (45.8→58.6 vs. 59.4→56.6) – TBARS (μM) ↓↓ (54.1→41.9 vs. 48.3→52.9) – TAC (mM) ↑↑ (2.1→2.9 vs. 2.5→2.3) – CAT (nmol/min/mL) ↑↑ (55.0→71.7 vs. 66.6→65.3) – SOD (U/mL) ↑↑ (1.7→2.0 vs. 2.3→2.3) – Glutathione (μM) ↑↑ (3.6→4.3 vs. 3.3→3.0)	No side effects reported
*Glycine max* (L.)/Genistein/Soy isoflavones						
Lohr et al., 2016 [65]	Open-label phase Ib clinical trial 13.2 months Jadad score: 4	N = 16 Patients with pancreatic carcinoma (metastatic or locally advanced, no prior treatment) M = 12; F = 4; Median age: 61 years (range 35–73)	Genistein/AXP107-11 (multi-component crystalline form of genisteine) – Escalating dose: 200 mg (three patients), 400 mg (three patients), 600 mg (three patients), 800 mg (seven patients) b.i.d. p.o. – Monotherapy for the first 2 weeks + Gemcitabine—1000 mg/m^2^/w during the first 7 of 8 weeks, then maximum of 4 × 4-week treatment cycles with dose given on days 1, 8, 15. Combined treatment for max. 6 months, then AXP107-11 monotherapy (0.4–7.2 months)	Pharmacokinetics (PK) Toxicity profile Maximum tolerated dose (MTD) Efficacy of AXP107-11 and Gemcitabine combination Response Time to progression (TTP) Progression-free survival (PFS) Overall survival (OS) CA19-9	– MTD not reached in the study. No more toxic events on lower or higher doses. Due to administration burden of large capsules, dose was stopped at 1600 mg/day = 16 capsules – PK: t-max 1.5 and 3 h; mean Cmax (800 mg AXP107-11) 1.1 μM; stable plasma concentrations, around 0.1 μM AXP107-11 (mean 0.07–0.14 μM) maintained 5–12 h post-dose – CA 19-9 ↓ of >50%: eight patients – Quality of life ↓ (70 vs. baseline 80–100, after average of 12 weeks (range 2–57)) – Stable disease (44%): seven patients – Partial responses (13%): two patients – Response duration 7.1 months: one patient – Progressive disease at first evaluation with short TTP: seven patients – >6-month survival: seven patients – 1-year survival: three patients (19%) – Median PFS 2.6 months (range 0.7–13.2) – Median OS 4.9 months (range 1.5–19.5 months)	No toxic adverse effects during AXP107-11 monotherapy (1st 2 weeks) – AXP107-11 did not increase toxicity in combination with Gemcitabine – Hematologic toxicities: – Grade 3 thrombocytopenia and platelet count, grade 4 neutropenia: one patient – Grade 3 neutropenia: three patients – Grade 3 non-hematological toxicities: four patients – Grade 3 white blood cell count: two patients – Non-hematologic toxicities: – Grade 3 fatigue, nausea: 1 patient – Grade 3 vomiting: one patient – Grade 3 infection: one patient – Grade 3 pancreatitis: one patient
El-Rayes et al., 2011 [66]	Open-label phase II clinical trial 23 months Jadad score: 4	N = 20 Patients with pancreatic adenocarcinoma (locally advanced unresectable or metastatic, no prior chemo-/radiotherapy) M = 12; F = 8; Median age: 58 years (range 39–75)	Soy isoflavones (Novasoy^®^: genistin, daidzin, glycitin in 1.3:1.0:0.3 ratio) – Dose: 531 mg (177 mg, three tablets) b.i.d. p.o. on day 7 until the end of study + Gemcitabine—dose 1000 mg/m^2^ i.v. on days 1, 8, 15 of each cycle + Erlotinib—150 mg s.d. p.o. on day 1–28 Cycles repeated every 28 days	Tumor response rate Progression-free survival (PFS) Overall survival (OS) Toxicity profile Immunohistochemistry for Akt and NF-κB	– Median OS: 5.2 months (95% CI, 4.6-N/A months) – Median PFS: 2 months (95% CI, 2.0–9.0 months) – 6-month survival rate: 50% (95% CI, 32–78%) – Partial response: one patient/5% – Stable disease: six patients/30% (95% CI, 12–54%) – Phosphorylated Akt, NF-kB grade 0–3 → grade 3—PFS: 4–14 weeks (however, no correlation proved to the best response, PFS) – No survival improvement by adding soy isoflavones to gemcitabine and erlotinib	– No toxicities due to soy isoflavones – Gemcitabine/Erlotinib related grade 3–4 toxicities: – Neutropenia: four patients – Thrombocytopenia: one patient – Nausea: one patient – Fatigue: five patients – Vomiting: three patients – Infection: one patient – Dehydration: one patient – Diarrhea: one patient – Grade 2 skin rash: 10 patients – Grade 3 cellulitis: one patient – DVT/pulmonary embolism: three patients
Sharma et al., 2019 [75]	Randomized placebo controlled clinical trial 60 days Jadad score: 3.5	N = 20 Patients with type 2 diabetes *Glycine max* group N = 10 Control group N = 10 Age range: 40–60 years	*Glycine max* leaves – Dose: 10 g of powder daily (incorporated in biscuits) Placebo: regular biscuits	Fasting blood glucose (FBG) Postprandial blood glucose (PBG) Glycated hemoglobin (HbA1c) Lipid profile: total cholesterol (TC), LDL, HDL, VLDL cholesterol, triglyceride	Mean change in *Glycine max* and placebo group, respectively (↓↓/↑↑ *p* < 0.05 in the intervention group, significant difference): – FBG (mg/dL) ↓↓ (−6.1 vs. 1.88) – PBG (mg/dL) ↓↓ (−9.23 vs. 0.76) – HbA1c (mg/dL) ↓↓ (−1.28 vs. 0) – TC (mg/dL) ↓↓ (−4.63 vs. 1.7) – triglyceride (mg/dL) ↓↓ (−8.8 vs. 2.2) – HDL (mg/dL) ↑ (0.95 vs. −0.57) – LDL (mg/dL) ↓↓ (−3.8 vs. 3.2) – VLDL (mg/dL) ↓↓ (−1.76 vs. 6.1)	No adverse effects reported
Choi et al., 2014 [76]	Randomized, double-blind placebo controlled clinical trial 12 weeks Jadad score: 6	N = 45 Patients with pre-type 2 diabetes *Glycine max* group N = 15 M = 7; F = 8; Mean ± SE age 49.71 ± 3.48 Control group N = 15 M = 9; F = 6; Mean ± SE age: 49.33 ± 4.15 Lagerstroemia speciosa (Banaba) group N = 15 M = 6; F = 9; Mean ± SE age: 47.00 ± 4.01	*Glycine max* (70% ethanol leaf extract, concentrated in vacuo and lyophilized to powder) – Dose: 1 g b.i.d. p.o. (2 g/day in total) Placebo (four capsules containing starch) – Dose: 2 g per day	Fasting blood glucose (FBG) Glycated hemoglobin (HbA1c) HOMA-IR (insulin resistance) Transaminase levels (AST, ALT) Total cholesterol (TC), LDL, HDL cholesterol Triglyceride Atherogenic Index (AI) Systolic, diastolic blood pressure (BP)	Mean baseline and post-treatment in *Glycine max* and placebo group, respectively (↓↓/↑↑ *p* < 0.05 in the intervention group, significant difference): – FBG (mg/dL) ↓↓ (112.00→102.50 vs. 119.43→124.57) – HbA1c (%) ↓↓ (6.35→6.14 vs. 6.191→6.73) – Plasma insulin (μL/U/mL) ↓ (3.92→3.62 vs. 3.70→3.89) – HOMA-IR ↓ (1.08→0.92 vs. 1.07→1.18) – Triglyceride (mg/dL) ↓ (262.88→217.13 vs. 269.83→269.93) – TC (mg/dL) ↓ (188.64→174.50 vs. 185.62→174.41) – HDL (mg/dL) ↑ (18.87→21.77 vs. 18.32→17.34) – LDL (mg/dL) ↓ (117.19→109.31 vs. 113.33→103.07) – AI ↓ (9.38→7.18 vs. 9.77→9.74) – ALT (U/L) ↓↓ (17.31→13.39 vs. 10.19→11.68) – AST (U/L) ↓↓ (25.15→23.55 vs. 17.92→21.06) – Systolic BP (mmHg) ↓ (129→124 vs. 128→125) – Diastolic BP (mmHg) ↑ (75.2→75.3 vs. 75.7→78.8)	No serious adverse effects reported
*Ginkgo biloba* L.						
Hauns et al., 1999 [67]	Open-label prospective phase II clinical trial Duration of evaluation—until progression Jadad score: 3.5	N = 32 Patients with pancreatic carcinoma (locally advanced or metastatic; prior treatment: surgery, chemo-/radio-/immunotherapy, none, other) M = 18; F = 14; Mean ± SD age: 58.2 ± 8.4	*Ginkgo biloba* (parenteral GBE 761 ONC/*Ginkgo biloba* leaves special extract EGb 761; one capsule 175 mg: 42 mg ginkgo flavone glycosides, 10.5 mg terpene lactones (ginkgolides, bilobalide) – Dose: 350 mg of GBE-761-ONC in 250 mL physiologic saline solution as 30 min infusion on days 1–6 + 5-fluorouracil – Dose: 500 mg/m^2^/day in 250 mL physiologic saline solution as 30 min. infusion on days 2–6 – Treatment cycle: every 3 weeks until progression	Tumor response rate Overall survival (OS) Efficacy Tolerability Toxicity profile Quality of life	– Progressive disease: 22 patients (68.8%) – Partial response: three patients (after four cycles) (9.4%) – Stable disease: seven patients (after four cycles) (21.9%) – Complete response: zero patients – Median OS: 5.6 months (range 2.6–7.3 months) – Disease duration > 15 months: one patients(3.1%) – Ginkgo + 5-FU combination—improvement of the treatment tolerability and overall quality of life – Quality of life—constant during the treatment period: – deterioration after first cycle: 10 patients; second cycle: 14 patients, third cycle: four patients; – Improvement after first cycle: 10 patients; second cycle: three patients; third cycle: seven patients; fourth cycle: three patients	All adverse events related to 5-FU, disease progression, other medication – Grade 3 leukopenia: three patients – Grade 3 thrombocytopenia, hemoglobin-related: two patients – Non-hematological toxicities: ↑ alkaline phosphatase, bilirubinemia (of grade 4): one patient – Gastrointestinal symptoms
Kudolo, 2001 [77]	Open-label follow-up controlled clinical trial 3 months Jadad score: 2.5	N = 20 Patients with type 2 diabetes Hyperinsulinemia group N = 12 – Diet-controlled N = 6 (M/F = 3/3; Mean ± SD age: 52 ± 6) – Hypoglycemic medication (metformin, glipizide, or glyburide) N = 6 (M/F = 4/2; mean ± SD age: 57 ± 6) Pancreatic exhaustion group N = 8 (M/F = 4/4; Mean ± SD age: 51 ± 9) on hypoglycemic medication (metformin, glipizide, glyburide, or troglitazone)	*Ginkgo biloba* (EGb 761—50:1 standardized *Ginkgo biloba* extract: 24% Ginkgo flavone glycosides, 6% terpenes) – Dose: 120 mg daily (administered to all participants)	Pancreatic β-cell function Fasting insulin (FI) Fasting C-peptide Fasting blood glucose (FBG) Fibrinogen Coagulation: Prothrombin time (PT), Partial thromboplastin time (PTT) Lipid profile (total cholesterol (TC), LDL, HDL cholesterol, triglyceride) Liver function (AST, ALT) Lactate dehydrogenase (LDH)	Mean baseline and post-treatment in hyperinsulinemic group of diet controlled and on medication and pancreatic exhaustion group, respectively (↓↓/↑↑ *p* < 0.05, significant difference): – FBG (mg/dL) 117→118 ↑ vs. 143→139 ↓ vs. 152→157 ↑ – FI (μU/mL) 29→26 ↓ vs. 46→39 ↓ vs. 16→20 ↑ – Fasting C-peptide (ng/mL) 3.8→3.7 ↓ vs. 5.2→4.4 ↓ vs. 2.5→3.3 ↑ – Fibrinogen (mg/dL) 286→287 ↑ vs. 345→343 ↓ vs. 307→336 ↑ – PT (s) 11.4→11.4 ø vs. 11.4→11.7 ↑ vs. 11.3→11.1 ↓ – PTT (s) 24.6→24.4 ↓ vs. 24.0→24.3 ↑ vs. 24.7→23.1 ↓ – TC (mg/dL) 194→186 ↓ vs. 176→159 ↓ vs. 181→183 ↑ – Triglycerides (mg/dL) 170→157 ↓ vs. 184→174 ↓ vs. 196→181 ↓ – HDL (mg/dL) 39→38 ↓ vs. 35→34 ↓ vs. 37→40 ↑ – LDL (mg/dL) 121→117 ↓ vs. 102→91 ↓ vs. 105→111 ↑ – AST (U/L) 23→23 ø vs. 30→30 ø vs. 23→22 ↓ – ALT (U/L) 28→29 ↑ vs. 31→31 ø vs. 30→23 ↓ – LDH (U/L) 173→153 ↓ vs. 184→163 ↓ vs. 105→152 ↑↑ – Response to glucose loading during a standard 75 g oral glucose – tolerance test: – Glucose area (mg/dL/h) 424→410 ↓ vs. 418→479 ↑↑ vs. 481→551 ↑ – Insulin area (μU/mL/h) 193→182 ↓ vs. 199→142 ↓↓ vs. 51→86 ↑↑ – C-peptide area (ng/mL/h) 14.3→15.9↑ vs. 15.6→15.1↓ vs. 7.2→13.7↑↑	No adverse effects reported – In contrast, effects reported by *Ginkgo biloba* use: – Increased wellbeing – Maintaining better mental focus – Increased sensation in the feet in patients with numbness
Aziz et al., 2018 [78]	Randomized double-blind placebo controlled clinical trial (multicenter) 90 days Jadad score: 6	N = 47 Patients with type 2 diabetes (Prior/current treatment: Metformin 500 or 850 mg) *Ginkgo biloba* group N = 27 M = 1; F = 26; Mean ± SD age 48.7 ± 9.6 Control group N = 20 M = 1; F = 19; Mean ± SD age 48.2 ± 10.3	*Ginkgo biloba* (extract as the standard powder (EGb761)) – Dose: 120 mg (capsule)/day + metformin 1.36 ± 0.45 g s.d. p.o. Placebo (starch) – Dose: 120 mg (capsule)/day + metformin 1.24 ± 0.67 g s.d. p.o .	Blood glycated hemoglobin (HbA1c) Fasting serum glucose (FSG) Serum insulin (SI) Body mass index (BMI) Insulin resistance (IR) Visceral adiposity index (VAI) Liver enzymes activity (AST, ALT, ALP) Urea Creatinine Hematocrit (Hct), Hemoglobin (Hb) Red/white blood cells Platelets	Mean baseline and post-treatment in *Ginkgo biloba* and placebo group, respectively, (↓↓/↑↑ *p* < 0.05 in the intervention group, significant difference): – HbA1c (%) ↓↓ (8.6→7.7 vs. 8.8→8.4) – FSG (mg/dL) ↓↓ (194.4→154.7 vs. 166.7→173.8) – SI (μU/mL) ↓↓ (18.5→13.4 vs. 17.5→15.8) – IR ↓↓ (9.0→N/A) vs. (9.4→N/A) – BMI kg/m2 ↓↓ (34.0→31.6 vs. no change (value N/A) – VAI ↓↓ (192.0→158.9 vs. 196.5→208.2) – AST (U/L) ↑ (18.4→18.8 vs. 22.2→18.1) – ALT (U/L) ↓ 17.8→17.5 vs. 19.2→17.0) – ALP (U/L) ↓↓ (93.8→86.1 vs. 97.6→82.1) – Urea (mg/dL) ↓↓ (28.1→24.5 vs. 25.6→27.1) – Creatinine (mg/dL) ↓↓ (0.69→0.60 vs. 0.67→0.73) – Hct (%) ↑↑ (37.1→41.0 vs. 38.3→40.9) – Hb (g/dL) ↑↑ (12.6→13.4 vs. 12.9→13.5) – RBC (×109 cells/L) ↑↑ (4.8→5.2 vs. 4.6→4.8) – WBC (×106 cells/L) ↓ (8.6→8.1 vs. 8.9→8.5) – Platelet (×109 cells/L) ↑ (233→242 vs. 252→206 ↓↓)	No serious adverse effects observed

↓/↑ decrease/increase of parameters; ↓↓/↑↑ significant decrease/increase

**Table 2:** Characteristics of clinical trials of low-carbohydrate ketogenic diet in the treatment of pancreatic adenocarcinoma and type 2 diabetes.

Author	Study Design, Duration, Quality	Participants (Sample Size, Diagnosis)	Intervention Preparation Used	Outcome Measures	Results	Adverse Events
Ok et al., 2018 [79]	Prospective controlled intervention study 6 months Jadad score: 4	N = 19 Patients with pancreatobiliary cancer after pancreatectomy (solely pancreatic cancer N = 6) Ketogenic diet group N = 10 M = 6; F = 4; Mean age: 59 years (range 49–70) Control group N = 9 M = 6; F = 3; Mean age: 66 years (range 54–79)	Ketogenic diet (KD) (3–6% energy as carbohydrates, 1 g/kg of protein, 70–80% of energy as fats→ketogenic ratio 1.05–1.75 (fat): 1 (carb + protein) General diet (carbohydrate:protein:fat (C:P:F) ratio—55–65:7–20:15–30	Dietary intake Meal satisfaction score Energy intake rate Lipid profile (total cholesterol (TC), HDL, LDL, total triglyceride (TG) C-reactive protein (CRP) Urine ketone (UK) Body composition (body weight (BW); body cell mass/BCM; body fat mass (BFM); skeletal muscle mass (SMM))	Mean ± SD values in KD and control group, respectively (↓↓/↑↑ *p* < 0.05 in the intervention group, significant difference): – Meal compliance (%) ↑↑ (69.1 ± 19.6 vs. 33.9 ± 16.6) – Energy intake rate (%) ↑↑ (61.3 ± 19.0 vs. 38.5 ± 21.9) – Overall meal satisfaction score ↑↑ (6.2 ± 1.8 vs. 3.8 ± 1.1) – Mean ± SD of baseline and post-treatment in KD and control group, respectively: – TC (mg/dL) ↓↓ (180.2 ± 145.7→173.8 ± 32.1 vs. 155.7 ± 46.1→156.0 ± 48.4) – HDL (mg/dL) ↓↓ (43.4 ± 9.7→38.4 ± 8.8 vs. 43.8 ± 11.2→42.4 ± 6.9) – LDL(mg/dL) ↓↓ (110.8 ± 37→108.4 ± 28.3 vs. 86.0 ± 36.5→97.4 ± 41.9) – TG (mg/dL) ↓ (131.8 ± 86.1→125.7 ± 49.8 vs. 120.7 ± 74.5→110.5 ± 51.1) – CRP (mg/L) ↑ (2.7 ± 3.7→25.6 ± 36.6 vs. 14.1 ± 35.0→10.0 ± 11.8) – UK (% of patients) ↑ (10→50 vs. 0→22.2) – BW (kg) ↓↓ (64.6 ± 11.2→60.6 ± 9.5 vs. 56.2 ± 7.2→52.7 ± 7.2) – BCM (kg) ↓↓ (28.9 ± 4.5→27.0 ± 5.0 vs. 27.4 ± 4.7→24.5 ± 4.2) – BFM (kg) ↓↓ (18.2 ± 5.2→17.1 ± 4.9 vs. 13.7 ± 6.2→14.2 ± 6.4) – SMM (kg) ↓↓ (24.3 ± 4.1→22.6 ± 4.5 vs. 23.0 ± 4.3→20.2 ± 3.8)	Frequency of meal intake-related adverse effects (percentage of patients) in KD and control group, respectively: – Anorexia ↓ (50.0 vs. 88.9) – Nausea ↓ (30.0 vs. 44.4) – Vomiting ↓ (10.0 vs. 33.3) – Constipation ↓ (0.0 vs. 11.1) – Abdomen pain ↑ (30.0 vs. 11.1) – Diarrhea ↓ (10.0 vs. 11.1) – No. of adverse events per person ↓ (1.3 ± 1.1 vs. 2 ± 1.2)
Tan-Shalaby et al., 2016 [80]	Open-label interventional phase I clinical trial 16 weeks Jadad score: 4	N = 11 Patients with solid cancer (advanced, metastatic, and unresectable; including pancreatic cancer N = 2; Other types: melanoma, brain, lung, prostate, renal, colon, head and neck, liver cancer; with prior treatment or without: four patients) M = 11; Mean age: 65 years (range 42–87) Two pancreatic adenocarcinoma patients: age 65 and 54 years	Ketogenic diet (modified Atkins diet; restriction on high carbohydrate foods/liquids e.g., cereal, bread, rice, pasta, potatoes, all fruits; no restriction on calories, protein or fats)	Toxicity profile Quality of life BMI, body weight Complete blood count Cholesterol profile Fasting glucose Serum ketone/beta-hydroxybutyrate (BHB) Serum creatinine Liver enzyme activity (ALT/alanine transferase) Tumor response rate	Mean ± SD of baseline and post-treatment, respectively (↓↓/↑↑ *p* < 0.05 in the intervention group, significant difference): – Weight loss (kg) ↓↓ (95 ± 18.7→87.7 ± 37.82) – BMI ↓↓ (30.3 ± 5.29→27.7 ± 4.69) – Fasting glucose (mg/dL) ↑ (89 ± 14.8→97.1 ± 12.07) – BHB ↓ (11.5 ± 10.27→9.14 ± 8.73) (Glucose/Ketone Index (GKI) for therapeutic benefit against cancer not reached) – Serum creatinine ↓ (1.14 ± 0.23→1.09 ± 0.21) – Total cholesterol ↓ (179 ± 34.6→186 ± 56.4) – LDL ↓ (125 ± 37.6→119 ± 40.76) – HDL ↑ (38.7 ± 15.09→46.75 ± 24.4) – Triglycerides ↓ (114 ± 44.45→103.58 ± 47.70) – WBC count ↑ (7.06 ± 4.84→7.17 ± 3.98) – ALT ↓ (28 ± 11.34→25 ± 13.78) Quality of life – Stable wellbeing with no significant deterioration Response rate (at 4 weeks): – Progressed disease: five patients – Stable or partly improved disease: six patients (→at 16 weeks: stable disease: four patients; reduced disease symptoms: one patient) Pancreatic adenocarcinoma patients: – First patient: weeks on trial/diet—four, progressive disease at 4 weeks – Second patient: weeks on trial/diet—N/A, very rapidly progressive disease, before starting diet	No significant adverse effects observed – Weight loss: eight patients/73% – Hyperuricemia: seven patients/64% – Hyperlipidemia, pedal edema, anemia, halitosis, pruritus, hypoglycemia, hyperkalemia, hypokalemia, hypomagnesemia, flu-like symptoms/fatigue: two patients/18%
Zahra et al., 2017 [81]	Open-label interventional phase I clinical trial 5 weeks (1 year follow-up) Jadad score: 4	N = 2 Patients with pancreatic cancer N = 1, F, age 69 years N = 1, M, age 67 years N = 7 Patients with non-small cell lung cancer	Ketogenic diet (KD) (4:1 ratio of fat(g): protein + carbohydrate(g); 90% of calories from fat, 8% from protein, 2% from carbohydrate) + Radiation (25 fractions of a total dose of 50 Gy) Chemotherapy (Gemcitabine 600 mg/m^2^ weekly, 5-FU)	Safety profile Ketone levels Blood glucose Oxidative stress Progression free survival (PFS) Overall survival (OS)	– One of two participants completed the study/KD with concurrent chemo-/radiotherapy. The second participant was removed from the study due to dose-limiting toxicity (grade 3 dehydration) – N = 1 → 50 Gy, Gemcitabine, ketosis duration 34 days (KD completed), weight loss 6.9 kg, Oxidative damage/protein carbonyl level ↑ Serum glucose ↓ Serum ketone levels ↑ Tumor progression, PFS 2 months, Secondary response—biliary obstruction and sepsis, OS 2 months – N = 1 → 50 Gy, Gemcitabine, 5-FU, ketosis duration 8 days (KD not completed), weight loss 9.4 kg, Tumor progression, PFS 5.3 months, Secondary response—ascites, OS 10 months	Treatment-related events and completed KD N = 1 – Grade 1–2 esophagitis, nausea, vomiting, constipation, diarrhea, concentration, thrombocytopenia, hypokalemia Not completed KD N = 1 – Grade 3 nausea, dehydration – Grade 1–2 vomiting, constipation, thrombocytopenia, hypokalemia – ↓ Compliance with KD during receiving concurrent chemo-/radiotherapy and related adverse events
Hagihara et al., 2020 [82]	Case series clinical study 3 months Jadad score: 4	N = 37 Patients with stage IV cancer, including pancreatic adenocarcinoma (Others: colorectal, non-small cell lung, breast, head and neck, bone and soft tissue, ovarian and peritoneal, endometrial, bladder, brain, biliary tract, gastric, prostate cancer) M = 15; F = 22; Mean age: 54.8 years Pancreatic adenocarcinoma patients (N = 4) M = 1 (age 48); F = 3 (age: 76, 74, 62 years) Prior treatment: Gemcitabine, Nab-Paclitaxel, S-1, FOLFIRINOX, Irreversible electroporation	Ketogenic diet (KD) – Week 1; Ketone ratio 2:1 – Carbs 10 g, Lipid 140 g, protein 60 g/day – Week 2–3 months; Ketone ration 2:1 to 1:1 – Carbs 20 g, Lipid 120–140 g, Protein 70 g – From month 3; Ketone ratio 1:1; Carbs 30 g (10 g/meal) – Caloric intake 30 kcal/kg/day + Calorie supplementation: medium chain triglyceride (MCT) oil (~50–80 g/day) and a ketogenic formula (~30 g/day)	Fasting blood glucose (FBG) β-hydroxybutyrate (BHB) Glucose ketone index (GKI) Tumor response rate Overall survival (OS) Safety profile	Baseline and post-treatment values in four patients with pancreatic cancer respectively (↓↓/↑↑ *p* < 0.05 significant difference): – FBG (mg/dL) ↓↓ 104→95; ↓↓ 121→119; ↑ 85→87; ↑↑ 97→112 – BHB (μmol/L) ↑↑229→1249; ↑↑308→1408; ↑↑19→3087; ↑↑417→949 – GKI ↓↓25.2→4.2; ↓↓21.8→4.7; ↓↓248.3→1.6; ↓↓12.9→6.6 – Median OS: 10.7 months – Overall results: – BHB ↑↑; FBG ↓↓; insulin levels ↓↓ – Serum albumin (Alb), C-reactive protein (CRP): no significant changes – GKI—moderate/functional to high ketosis: 70% of patients – Partial response: five patients at 3 months, seven patients at 1 year – Complete response: zero patients at 3 months, three patients at 1 year – Stable disease: 19 patients at 3 months, eight patients at 1 year – Progressive disease—13 patients at 3 months, 11 patients at 1 year – Response rate (partial + complete): 27% – Median OS: 32.2 months (max 80.1) – 3-year survival rate: 44.5%	868 adverse events related to chemotherapy/disease progression (all cancers) 275 events related to KD Grade 1–2 adverse events: – Hyperuricemia (58.2%) – Hyperlipidemia (52.7%) – High cholesterol (45.5%) – Constipation (30.9%) – Weight loss (21.8%) – Hypertriglyceridemia (23.6%) – Hypoglycemia (18.2%) – Stomach pain (14.5%) – Diarrhea (12.7%) – Hypokalemia (9.1%) – Abdominal pain (3.6%) – Hypocalcemia, vomiting, nausea, dyspepsia, anorexia, muscle cramp, malaise (1.8%) Grade 3 adverse events: – High cholesterol (1.8%)
Westman et al., 2008 [83]	Open-label randomized controlled clinical trial 24 weeks Jadad score: 7.5	N = 50 Patients with type 2 diabetes Low-carbohydrate ketogenic diet group N = 21 M = 7; F = 14; Mean ± SD age: 51.2 ± 6.1 Control/low-glycemic diet group N = 29 M = 6; F = 23; Mean ± SD age: 50 ± 8.4 (Prior treatment: insulin, metformin, rosiglitazone, pioglitazone, glimepiride)	Low-carbohydrate ketogenic diet (LCKD) (<20 g of carbohydrate/day without explicit caloric restriction; unlimited amounts of animal foods, limited amounts of cheese and vegetables) Low-glycemic reduced-calorie diet (LGID) (~55% carbohydrate; energy intake 500 kcal—less than calculated energy intake for weight maintenance) – Recommendation: exercise for 30 min at least three times a week – Nutritional supplements: vanadyl sulfate 200 mcg/day, chromium dicotinate glycinate 600 mcg/day, alpha-lipoic acid 200 mg/day	Glycosylated hemoglobin (HbA1c) Fasting glucose (FG) Fasting insulin (FI) BMI Body weight (BW) Lipid profile (total cholesterol (TC), HDL, LDL, triglycerides, VLDL) Blood pressure (BP) Diabetes medication use	Mean change in KD and control group, respectively (↓↓/↑↑ *p* < 0.05 within groups, * between groups; significant difference): – HbA1c (%) ↓↓ (−1.5 vs. −0.5), * – FG (mg/dL) ↓↓ (−19.9 vs. −16.0) – FI (μU/mL) ↓↓ (−6.0 vs. −2.2) – BMI (kg/m2) ↓↓ (−3.9 vs. −2.7), * – Body weight (kg) ↓↓ (−11.1 vs. −6.9), * – Triglycerides (mg/dL) ↓↓ (−67.5 vs. −19.3) – HDL (mg/dL) ↑↑ (5.6 vs. −0), * – LDL (mg/dL) ↑↓ (1.3 vs. −2.8) – TC (mg/dL) ↓ (−4.4 vs. −5.8) – VLDL (mg/dL) ↓↓ (−10.0 vs. −3.3) – Systolic BP (mmHg) ↓↓ (−16.6 vs. −10.7) – Diastolic BP (mmHg) ↓↓ (−8.1 vs. −5.6) – Serum creatinine and calculated GFR: no significant change for either group – Urine protein (mg/24 h) ↓↓ (445 ± 1175→296 ± 750 vs. 276 ± 705→ 223 ± 623); * – Diabetes medications use ↓↓/eliminated in 95.2% of LCKD vs. 62% of LGID patients	No serious adverse effects reported – Headache (LCKD: 53.1%, LGID: 46.3%) – Constipation (LCKD: 53.1%, LGID: 39.0%) – Diarrhea (LCKD: 40.6%, LGID: 36.6%) – Insomnia (LCKD: 31.2%, LGID: 19.5%) – Back pain (LCKD: 34.4%, LGID: 39.0%)
Hallberg et al., 2018 [84]	Open-label, non-randomized interventional controlled clinical trial 1 year Jadad score: 3.5	N = 296 Patients with type 2 diabetes Continuous care intervention (CCI) group N = 218 M = 76; F = 142; Mean ± SD age: 54 ± 8 (92% obese, 88% on diabetic medication) Usual care (UC) group N = 78 M = 31; F = 47; Mean ± SD age: 52 ± 10 (82% obese, 87% on diabetic medication) Prior/current medication: metformin, insulin, sulfonylurea, thiazolidinedione, sodium-glucose cotransporter-2 (SGLT2) inhibitors, dipeptidyl peptidase 4 (DPP-4) inhibitors, glucagon-like peptide-1 receptor (GLP-1) agonists	Ketogenic diet (KD) (Instructions: carbohydrates <30 g/day; protein < 1.5 g kg−1 of reference body weight; fats- the rest of dietary intake; 3–5 servings of non-starchy vegetable) Recommendation: 1000–2000 IU vitamin D3, up to 1000 mg omega-3/day; 500 mg magnesium oxide or 200 mg magnesium chloride (if magnesium depletion); nutritional ketosis goal: 0.5–3.0 mmol L-1 blood BHB (β-hydroxybutyrate)	Glycated hemoglobin (HbA1c) Weight Medication use Fasting serum glucose (FSG) Fasting serum insulin (FSI) HOMA-IR Lipid/lipoprotein profile (total cholesterol (TC), LDL, HDL, Apo B, Triglyceride) High-sensitivity C-reactive protein (hs-CRP) Liver function (ALT, AST, ALP) Kidney function (serum creatinine, blood urea nitrogen (BUN))	Mean change ± SE in CCI vs. UC group, respectively (↓↓/↑↑ *p* < 0.05 in the CCI vs. UC group, significant difference): – HbA1c (%) ↓↓ (−1.32 ± 0.09 vs. 0.22 ± 0.16), −17% in CCI – BHB (μmol/L) ↑ (0.13 ± 0.02 vs. 0.06 ± 0.05) – FSG (mg/dL) ↓↓ (−2.02 ± 0.26 vs. 0.81 ± 0.45), −22% in CCI – FSI (pmol/L) ↓↓ (−91.4 ± 12.15 vs. 36.88 ± 29.66), −43% in CCI – C-peptide (nmol/L) ↓↓ (−0.34 ± 0.05 vs. 0.02 ± 0.09), −23% in CCI – HOMA-IR (serum insulin derived) ↓↓ (−6.13 ± 0.98 vs. 4.1 ± 2.34), −55% in CCI – HOMA-IR (C-peptide derived) ↓↓ (−3.53 ± 0.55 vs. 1.77 ± 1.12), −29% in CCI – Weight (kg) ↓↓ (−13.81 ± 0.63 vs. −1.11 ± 1.06), −12% in CCI – TC (mmol/L) ↑ (0.24 ± 0.08 vs. 0.0 ± 0.16) – LDL (mmol/L) ↑↑ (0.28 ± 0.07 vs. −0.28 ± 0.13), +10% in CCI – HDL (mmol/L) ↑↑ (0.19 ± 0.02 vs. −0.02 ± 0.04), +18% in CCI – Apo B (g/L) ↓/ø (0.0 ± 0.02 vs. 0.0 ± 0.04) – Triglycerides (mmol/L) ↓↓ (−0.56 ± 0.18 vs. −0.35 ± 0.32), −24% in CCI – hs-CRP (nmol/L) ↓↓ (−29.43 ± 9.14 vs. 8.48 ± 16.1), −39% in CCI – ALT (μkatL) ↓↓ (−0.16 ± 0.03 vs. 0.02 ± 0.05), −30% in CCI – AST (μkatL) ↓↓ (−0.09 ± 0.02 vs. 0.01 ± 0.04), −21% in CCI – ALP (μkatL) ↓↓ (−0.17 ± 0.02 vs. 0.02 ± 0.03), −13% in CCI – Serum creatinine (μmol/L) ↓ (↓↓−3.54 ± 0.88 vs. −2.65 ± 1.77) – BUN (mmol/L) ↑↑ (0.75 ± 0.17 vs. 0.06 ± 0.3) – All diabetes medication use, exl. Met(%) ↓↓ (−27.66 ± 3.21 vs. 7.54 ± 5.87) – Insulin medication use (%) ↓↓/stopped (−15.5 ± 2.0 vs. 8.46 ± 3.65)—94% users – Metformin use (%) ↓↓ (−7.14 ± 3.0 vs. 0.83 ± 5.5) – Sulfonylureas ↓ (eliminated)—100% of users UC group: no significant changes in biomarkers or T2D medication	No serious adverse events reported in the CCI – Significant ↑ in mean blood urea nitrogen, probably due to high protein intake, even though not recommended – Subclinical hypothyroidism: two patients – Adverse events in six/87 UC patients, not attributable to the intervention – Percutaneous coronary intervention (PCI) to left anterior descending stenosis – PCI to right coronary artery – Carotid endarterectomies due to carotid artery disease – Multifactorial encephalopathy – Diabetic ketoacidosis with pulmonary emboli
Myette-Côté et al., 2018 [85]	Open-label randomized crossover controlled clinical trial Three 4-day interventions with a washout period of 9–14 days between interventions Jadad score: 6.5	N = 11 Patients with type 2 diabetes M = 4; F = 7; Mean ± SD age: 64 ± 8 Low-carbohydrate, high-fat diet/ketogenic diet group (LC) LC with post-meal walks group (LC + Ex) Low-fat low-glycemic index diet group (GL) Prior treatment: metformin, sulfonylurea, glucagon-like-peptide-1, dipeptidyl peptidase-4, statin, antihypertensive >3 days of structured exercise per week	Low-carbohydrate high-fat diet/ketogenic diet (LC) (carbohydrate 10%, protein 25%, fat 65%; saturated fat 15%, polyunsaturated fat 11%, monounsaturated fat 39%) LC with 15-min 3 daily post-meal walks (LC + Ex) Low-fat low-glycemic index diet (GL) (carbohydrate 55%, protein 25%, fat 20%; saturated fat 5%, polyunsaturated fat 5%, monounsaturated fat 10%; mean glycemic index: 40)	Fasting glucose (FG) Triglycerides Fasting insulin (FI) Active proinsulin C-peptide Inflammatory markers (tumor necrosis factor-α/TNF-α, monocyte chemoattractant protein-1/MCP-1, interleukin-6 (IL-6), IL-10, IL-18) Monocyte and leukocyte-derived microparticles (MMPs, LMPs) p-JNK/phosphorylated c-Jun NH 2 -terminal kinase Toll-like receptor (TLR, median fluorescence intensity) Granulocytes (Gr), Lymphocytes (Ly), Monocytes (Mo)	Mean baseline and post-treatment in LC, LC + Ex, GL group, respectively (↓↓/↑↑ *p* < 0.05 in the LC/LC + Ex group, significant difference): – FG (mmol/L) ↓↓ (8.4→7.6 vs. ↓↓ 7.8→7.0 vs. 8.3→8.1) – Triglycerides (mmol/L) ↑ (1.9→2.1 vs. 1.9→1.9 vs. ↓ 2.0→1.9) – FI (pmol/L) ↓ (64.8→62.1 vs. 59.6→50.1 vs. 63.9→58.0) – Proinsulin (pmol/L) ↓↓ (35.5→26.0 vs. ↓↓ 35.3→22.8 vs. 33.5→30.7) – C-peptide (nmol/L) ↓ (1.18→1.07 vs. 1.20→1.05 vs. 1.11→1.18) – Proinsulin-insulin ratio ↓ø (0.7→0.6 vs. 0.6→0.6 vs. 0.7→0.6) – Proinsulin-C-peptide ratio ↓↓ (0.03→0.025 vs. 0.032→0.022 vs. 0.031→0.027) – TNF-α (pg/mL) ↑ (14.1→14.8 vs. 14.8→15.7 vs. 14.6→15.7) – MCP1 (pg/mL) ↓↓ (727→686 vs. ↓↓ 769→680 vs. 725→710) – IL-6 (pg/mL) ↑ (8.2→8.8 vs. ↓ 9.9→9.2 vs. 8.1→9.1) – IL-18 (pg/mL) ↓ (2838→2818 vs. 2691→2658 vs. 2810→2760) – IL-10 (pg/mL) ↓ (3.5→3.3 vs. ø 3.8→3.8 vs. 3.6→3.3) – p-JNK (A.U./arbitrary units) ↓↓ (105→58 vs. ↓↓73→41 vs. ↓↓ 100→68) – TLR2 ↓ (7.4→6.9 vs. 7.8→7.7 vs. 7.5→6.9) – TLR4 ↑ ø (5.0→5.0 vs. ↑ 5.0→5.2 vs. 4.7→4.8) – MMPs (count/mL) ↓↓ (245→73 vs. 238→239 vs. ↓↓ 404→97) – LMPs (count/mL) ↓ (1055→890 vs. 620→565 vs. 1313→490) – Gr (count/mL × 106) ø↓ (2.6→2.6 vs. ↓ 2.9→2.5 vs. 2.4→2.9) – Ly (count/mL × 106) ø↓ (1.3→1.3 vs. 1.3→1.2 vs. 1.2→1.4) – Mo (count/mL × 105) ↑↓ (2.6→2.7 vs. 3.0→2.6 vs. 2.7→3.0)	Not provided
Saslow et al., 2017 [86]	Interventional randomized controlled clinical trial 32 weeks Jadad score: 7	N = 25 Patients with type 2 diabetes Ketogenic diet group N = 12 M = 6; F = 6; Mean ± SD age: 53 ± 10.2 Control group N = 13 M = 4; F = 9; Mean ± SD age 58.2 ± 6.7 (Prior medication: none or metformin)	Very low-carbohydrate ketogenic diet (KD), (20–50 g carbohydrate daily) + Lifestyle recommendation (mental wellbeing, physical activity, sleep) “Create your plate” diet (low-fat diet; half a plate of nonstarchy vegetables; quarter plate of carbohydrates; quarter plate of lean proteins)	Glycated hemoglobin (HbA1c) Body weight (BW) Lipid profile (triglycerides, HDL, LDL) Diabetes-related distress Depression Vitality	Mean change in KD/intervention and control group, respectively (↓↓/↑↑ *p* < 0.05, between groups, significant difference): – HbA1c (%) ↓↓ (−0.8 vs. −0.3) – Weight (kg) ↓↓ (−12.7 vs. −3.0) – Triglycerides (mg/dL) ↓↓ (−60.1 vs. −6.2) – HDL (mg/dL) ↑ (4.8 vs. 0.6) – LDL (mg/dL) ↓ (−0.3 vs. −6.1) – Diabetes-related distress ↓ (−0.4 vs. −0.4) – CES-Depression ↓ (−0.6 vs. −1.0) – Vitality (SF-36 subscale) ↑ (9.2 vs. 11.0) – HbA1c <6.5% (% of patients) (55 vs. 0; *p* = 0.02) – Weight (% of initial weight) (mean) (−12.0 vs. −2.5; *p* = 0.01) – Achieving a 5% weight loss (%) (90% vs. 29%; *p* = 0.01) – Metformin use in KD group: ↓ one patient, ↑ two patients, ø eight patients – Metformin use in control group: ↓ two patients, ↑ one patient, ø four patients	Not provided Physical self-report: – Headache, bloating, gas ↓ in the KD group vs. control – Constipation ↑ in the KD group vs. control
Forsythe et al., 2008 [87]	Interventional randomized controlled clinical trial 12 weeks Jadad score: 4	N = 40 Patients with type 2 diabetes Age: 18–55 years Ketogenic diet group N = 20 Control group N = 20	Very low carbohydrate ketogenic diet (VLCKD) (1504 kcal: % carbohydrate:fat:protein = 12:59:28) Low fat diet (LFD) (1478 kcal: % carbohydrate:fat:protein = 56:24:20) (the multivitamin /mineral complex giving micronutrients at levels ≤100% of the recommended dietary allowance)	– Lipid profile (total cholesterol (TC), HDL, LDL, triglycerides, phospholipids) – Inflammatory markers: IL-6,8, Vascular endothelial growth factor (VEGF), TNF-a, Interrferon (IFN-c), Epidermal growth factor (EGF), Monocyte chemotactic protein-1 (MCP-1), Intracellular cellular adhesion molecule-1 (ICAM-1), Vascular cellular adhesion molecule-1 (V-CAM-I), E-selectin, P-selectin, L-selectin, C-reactive protein (CRP) – Plasminogen activator inhibitor-1 (PAI-1) – Immune cells profile (white blood cells (WBC), neutrophils, lymphocytes, monocytes, eosinophils, basophils)	Mean baseline and post-treatment in KD and control group, respectively (↓↓/↑↑ *p* < 0.05 within intervention/KD group, * between groups, significant difference): – TC (mg/dL) ↓↓ (208.0→196.5 vs. 204.0→194.5) – LDL (mg/dL) ↓ (130.4→135.4 vs. 127.9→125.9) – HDL (mg/dL) ↑↑ (35.8→40.4 vs. 38.7→38.4), * – Triglycerides (mg/dL) ↓↓ (210.9→103.7 vs. 187.1→151.2), * – Phospholipids (mg/dL) ↓↓ (196→170 vs. 183→173) – CRP (mg/dL) ↓↓ (0.6→0.5 vs. 0.4→0.3) – IL-6 (pg/mL) ↓ (8.4→5.5 vs. 6.3→6.3) – IL-8 (pg/mL) ↓↓ (8.5→5.7 vs. 9.4→9.8), * – VEGF (pg/mL) ↓↓ (162→122 vs. ↑ 129→130) – TNF-a (pg/mL) ↓↓ (2.8→1.9 vs. 2.6→2.3), * – IFN-c (pg/mL) ↓ (2.2→1.7 vs. 2.0→2.2) – EGF (pg/mL) ↓ (12.6→6.9 vs. 20.7→13.7) – MCP-1 (pg/mL) ↓↓ (380→288 vs. 323→307), * – I-CAM (ng/mL) ↓↓ (360→299 vs. 338→328), * – V-CAM (ng/mL) ↓ (549→512 vs. 567→536) – E-selectin (ng/mL) ↓↓ (18.9→12.4 vs. 16.7→14.4), * – P-selectin (ng/mL) ↓↓ (125→107 vs. 122→112) – L-selectin (ng/mL) ↓ (1148→1091 vs. ↑ 1081→1098) – PAI-1 (ng/mL) ↓↓ (45.0→29.5 vs. 38.3→35.2), * – WBC (×109/L) ↓ ø (6.2→5.9 vs. 5.9→5.9) – Neutrophils (×109/μL) ↓ (3507→3460 vs. 3368→3428) – Lymphocytes (×109/μL) ↓ (2039→1741 vs. 1899→1892) – Monocytes (×109/μL) ↑ (402→451 vs. ↓506→422) – Eosinophils (×109/μL) ↓ (210→176 vs. 184→164) – Basophils (×109/μL) ↓ (26→21 vs. ↑ 24→27)	Not provided

↓/↑ decrease/increase of parameters; ↓↓/↑↑ significant decrease/increase; * significant difference between groups

## Data Availability

Not applicable.
